# Analysis of m^6^A-related lncRNAs for prognostic and immunotherapeutic response in hepatocellular carcinoma

**DOI:** 10.7150/jca.92128

**Published:** 2024-02-17

**Authors:** Xingwei Wu, Shengnan Wang, Xiaoming Wu, Qianyi Chen, Jin Cheng, Zhilin Qi

**Affiliations:** 1 Department of Biochemistry and Molecular Biology, Wannan Medical College, Wuhu, Anhui 241002, P.R. China.; 2 Anhui Province Key Laboratory of Active Biological Macro-molecules, Wannan Medical College, Wuhu, Anhui 241002, P.R. China.; 3 Clinical Laboratory, Traditional Chinese Hospital of Lu'an, Anhui University of Chinese Medicine, Lu'an 237000, Anhui, P.R. China.; 4 Department of Pathology, Fuyang People's Hospital, Anhui Medical University, Fuyang, Anhui, 236000, P.R. China.; 5 Department of Thyroid and Breast Surgery, Yijishan Hospital, First Affiliated Hospital of Wannan Medical College, Wuhu, Anhui, 241002, P.R. China.; 6 Department of Gastroenterology, Yijishan Hospital, First Affiliated Hospital of Wannan Medical College, Wuhu, Anhui, 241002, P.R. China.

**Keywords:** m^6^A, lncRNAs, HCC, LASSO, immune

## Abstract

**Background:** RNA methylation modifications are important post-translational modifications that are regulated in an epigenetic manner. Recently, N^6^-methyladenosine (m^6^A) RNA modifications have emerged as potential epigenetic markers in tumor biology.

**Methods:** Gene expression and clinicopathological data of LIHC were obtained from the cancer genome atlas (TCGA) database. The relationship between long non-coding RNAs (lncRNAs) and m^6^A-related genes was determined by gene expression analysis using Perl and R software. Co-expression network of m^6^A-lncRNA was constructed, and the relevant lncRNAs associated with prognosis were identified using univariate Cox regression analysis. These lncRNAs were then divided into two clusters (cluster 1 and cluster 2) to determine the differences in survival, pathoclinical parameters, and immune cell infiltration between the different lncRNA subtypes. The least absolute shrinkage and selection operator (LASSO) was carried out for regression analysis and prognostic model. The HCC patients were randomly divided into a train group and a test group. According to the median risk score of the model, HCC patients were divided into high-risk and low-risk groups. We built models using the train group and confirmed them through the test group. The m^6^A-lncRNAs derived from the models were analyzed for the tumor mutational burden (TMB), immune evasion and immune function using R software. AL355574.1 was identified as an important m^6^A-associated lncRNA and selected for further investigation. Finally,* in vitro* experiments were conducted to confirm the effect of AL355574.1 on the biological function of HCC and the possible biological mechanisms. Huh7 and HepG2 cells were transfected with AL355574.1 siRNA and cell proliferation ability was measured by CCK-8, EdU and colony formation assays. Wound healing and transwell assays were used to determine the cell migration capacity. The expression levels of MMP-2, MMP-9, E-cadherin, N-cadherin and Akt/mTOR phosphorylation were all determined by Western blotting.

**Results:** The lncRNAs with significant prognostic value were classified into two subtypes by a consistent clustering analysis. We found that the clinical features, immune cell infiltration and tumor microenvironment (TME) were significantly different between the lncRNA subtypes. Our analysis revealed significant correlations between these different lncRNA subtypes and immune infiltrating and stromal cells. We created the final risk profile using LASSO regression, which notably included three lncRNAs (AL355574.1, AL158166.1, TMCC1-AS1). A prognostic signature consisting of the three lncRNAs was constructed, and the model showed excellent prognostic predictive ability. The overall survival (OS) of the low-risk cohort was significantly higher than that of the high-risk cohort in both the train and test group. Both risk score [hazard ratio (HR)=1.062; P<0.001] and stage (HR=1.647; P< 0.001) were considered independent indicators of HCC prognosis by univariate and multivariate Cox regression analysis. In Huh7 and HepG2 cells, AL355574.1 knockdown inhibited cell proliferation and migration, suppressed the protein expression levels of MMP-2, MMP-9, N-cadherin and Akt/mTOR phosphorylation, but promoted the protein expression levels of E-cadherin.

**Conclusions:** This study established a predictive model for the OS of HCC patients, and these OS-related m^6^A-lncRNAs, especially AL355574.1 may play a potential role in the progression of HCC. *In* v*itro* experiments also showed that AL355574.1 could enhance the expression of MMPs and EMT through the Akt/mTOR signaling pathway, thereby affected the proliferation and migration of HCC. This provides a new perspective on the anticancer molecular mechanism of AL355574.1 in HCC.

## Introduction

HCC, the most lethal global malignancy, is diagnosed in more than 90% of liver cancer cases^1^. Currently, available treatments for HCC, including surgery, chemotherapy, radiotherapy, targeted therapy and biological therapy. The lack of symptoms in the early stages of the disease leads to a late diagnosis and often to a poor outcome of the treatment.^2^. Therefore, it is urgent to search for diagnostic biomarkers and identify HCC therapeutic strategies.

lncRNAs are non-protein coding RNA fragments exceeding 200 bp in length^3^. Based on their genomic loci, lncRNAs were classified into antisense lncRNAs, intronic non-coding RNAs, intergenic lncRNAs, sense lncRNAs and bidirectional lncRNAs^4^. LncRNAs play crucial roles in cancer progression via various mechanisms, including chromosome remodeling, chromatin interactions, ceRNA machinery and natural antisense transcripts (NATs)^5^-^8^. Recent study have shown that lncRNAs were recognized as important players in the promotion of cancer and act as oncogenes during tumor development^9^. Due to their abundance and specific expression, lncRNAs are implicated in multiple types of cancer, affecting the biological functions of tumor cells, and eventually leading to tumorigenesis. For example, lncRNA FGD5-AS1 accelerates the proliferation of pancreatic cancer cells by regulating miR-520a-3p/KIAA1522 axis^10^, lncRNA MIR22HG regulates the proliferation and apoptosis of numerous human cancers^11^. In addition, aberrations in lncRNA expression, deletion or mutation are closely associated with HCC development and metastasis, indicating their potential as oncogenes^12^.

Beyond established DNA and histone modifications, mRNA modifications may also play an important role in tumor pathogenesis^13^. m^6^A is an adenine (A) methylation modification found in RNA. Many eukaryotic mRNAs and lncRNAs contain this sequence. Abnormal m^6^A modifications can stimulate tumor stem cell self-renewal, thus promoting tumor development^14^. Therefore, restoring RNA methylation in tumor cells may be a novel anti-cancer strategy^15^. The m^6^A methylation-related meth lest erases and demethylases can be classified into three types according to their distinct function. The enzymes, act as m^6^A “writers”, such as METTL3, METTL14 and WTAP, assemble their subunits into m^6^A complexes^16^. The proteins that recognize methylation sites are called “readers”, that include YTHDC1, YTHDC2, YTHDF1, YTHDF2, and HNRNPC. Proteins with the YTH domain bind to methylated mRNA specifically and regulate downstream translation and degradation^17^. “Erasers”, including FTO and ALKBH5, can demethylate RNA. FTO has the similar structure with ALKBH5 in core domain and is closely related to cancer^18^. Recently, the interplay between m^6^A methylation and lncRNAs in tumors has attracted considerable attention, and m^6^A-associated lncRNAs have been proposed as potential prognostic targets in various cancers^19^. This study specifically explores lncRNA-based m^6^A methylation modifications with HCC.

**Figure 1:**
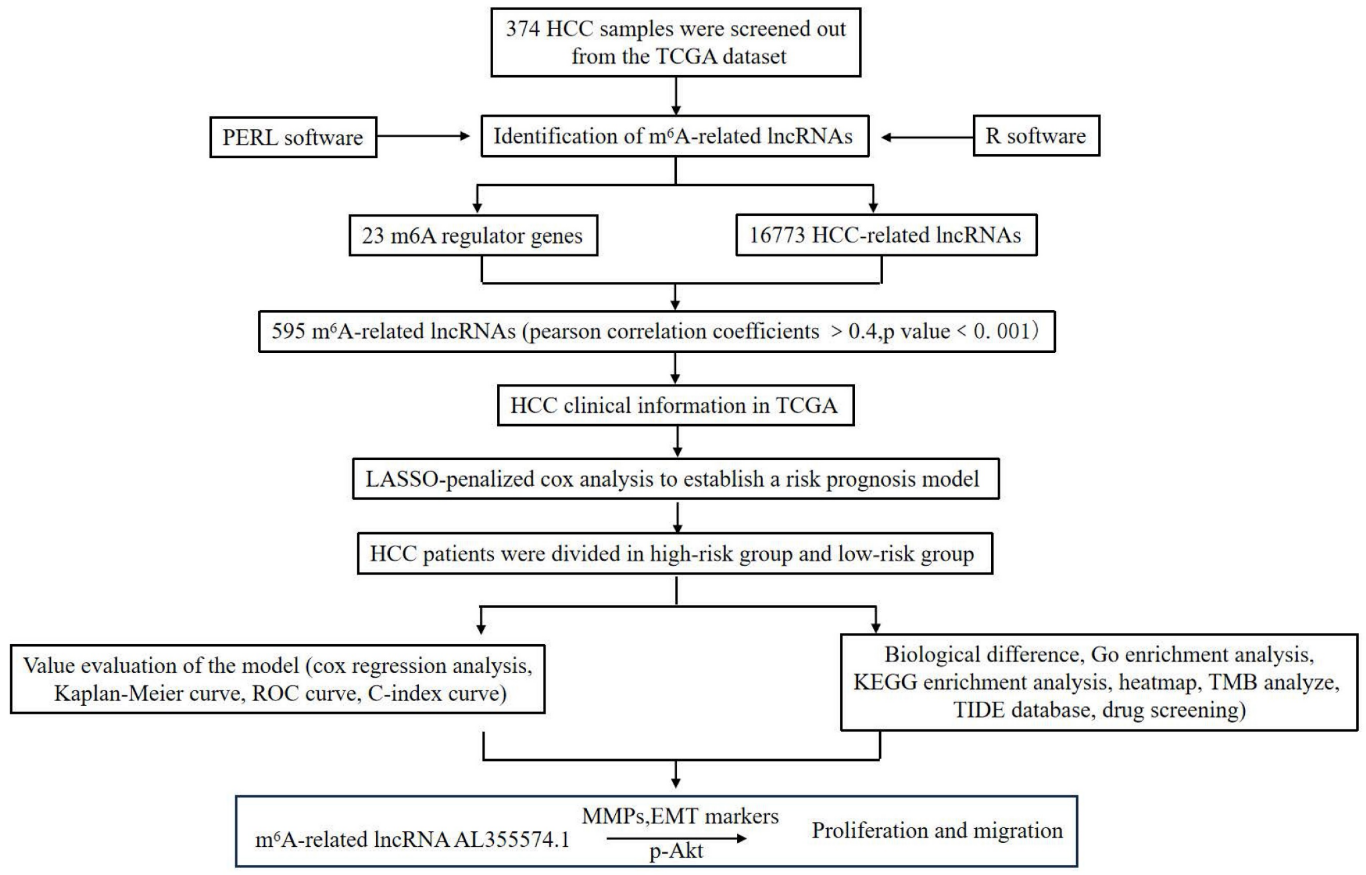
Workflow of the study.

This study aimed to screen m^6^A-related lncRNAs in HCC and comprehensively analyse their potential roles in clinical features, prognosis and TME. We also attempted to construct a prognostic prediction model and explore possible molecular markers and drug targets. This study will provide new research strategies for HCC immunotherapy. The methodology employed in this investigation is visually depicted in Figure 1.

## Materials and methods

### Sample data collection

The TCGA database was used to download gene expression and clinical data from the LIHC dataset. The mRNA expression matrices were obtained by extracting the expression profiles of 50 normal samples and 374 HCC tissue samples. Meanwhile, clinical data such as survival data, gender, age, histological grade, pathological stage and TMN stage of HCC patients were collected. Then, m^6^A-related genes were analysed using R software. The expression data of m^6^A gene-related lncRNAs were obtained by co-expression analysis of lncRNA and m^6^A-related gene expression. The expression data of m^6^A-related lncRNAs were combined with clinical survival data using the "limma" package. Prognostically relevant lncRNAs were extracted and confidence intervals and hazard ratios were calculated using the “survival” package. Differences in m^6^A-related prognostic lncRNA expression between tumor tissues and normal tissues were determined using the "limma" package, the "pheatmap" package, the "reshape" package and the "ggpubr" package, P<0.05 was considered statistically significant. We identified a total of 19,819 protein-coding genes and 16,200 lncRNAs by analysing the TCGA-LIHC dataset. The pearson correlation coefficients between the 23 m^6^A-related protein-coding genes and 16,200 lncRNAs were calculated using the COR-test function built into the R software. P<0.0001 was then selected for the screening process. Finally, 23 differentially expressed m^6^A-associated lncRNAs with prognostic value were retained for further analysis. A total of 23 common m^6^A-associated genes were identified in the literature, including "Writers" (METTL3, METTL14, METTL16, WTAP, VIRMA, ZC3H13, RBM15 and RBM15B); “Readers" (YTHDC1, YTHDC2, YTHDF1, YTHDF2, YTHDF3, HNRNPC, FMR1, LRPPRC, HNRNPA2B1, IGFBP1, IGFBP2, IGFBP3 and RBMX); "Erasers" (FTO and ALKBH5).

### The function analysis of m^6^A-associated lncRNAs

The "consensusdusterplus" and "limma" packages were used to classify the prognostic m^6^A-lncRNAs into two subtype groups (cluster 1 and cluster 2) based on the lncRNA expression using clusterAlg=km and clusterNum=2. The clinicopathological parameters of the patients was analyzed according to the lncRNA subtype group using the "Survminer" package. The "pheatmap" package was used to create a heatmap. Correlation between target genes and m^6^A-lncRNA prognosis was analyzed using the "limma" package. Differences were considered statistically significant when p<0.05.

### Function analysis of m^6^A-related lncRNA in immune infiltration and TME

To analyse and calculate the infiltration of different immune cells in tissue samples, the "estimate" and "limma" packages for TME were used to obtain the StromalScore, ImmuneScore and ESTIMATEScore. Each immune cell infiltration in different lncRNA subtypes was also analysed using the "limma" package, which was described using a boxplot.

### M^6^A-related lncRNA gene marker modelling and evaluation

The integrated TCGA-LIHC dataset was randomly divided into train and test group. The train group was used to build the m^6^A-associated lncRNA model. The test group was used to validate the model and present the baseline characteristics of the train and test group. There was no significant difference (P>0.05) in the clinical characteristics of these two datasets. A risk score (RS) was calculated for each sample to construct a prognostic model. Cox analyses were performed based on the clinical data and RS of HCC patients. The C-index was calculated to assess the best predictive outcome of the model. Based on median risk score, the train and test group were divided into high-risk and low-risk groups. ROC curves were generated using the "survival" package in the R software to assess the accuracy of the genetic characteristics in predicting survival. The "Heatmap" shows the prognostic lncRNA heatmaps for the train and test group.

### Identification the independence of the m^6^A-associated lncRNA model

Univariate and multivariate Cox regression analyses were performed to test whether the prognostic model was independent of other clinical characteristics (age, gender and pathological stage) in HCC patients.

### Investigation of models in immunotherapy

Mutation data were calculated and evaluated using the R package maftools. TMB was measured based on tumor-specific mutated genes. The TIDE algorithm was used for prediction of the likelihood of response to immunotherapy.

### Prinicipal Component Analysis (PCA) analysis

PCA analysis was used to reduce the dimensionality of large gene expression data and to perform hierarchical clustering of all samples. 3D scatterplot was also used to visualise the distribution of all samples. Efficient dimensionality reduction of the whole gene expression profile, the m^6^A genes and the m^6^A-associated lncRNAs. Based on the expression pattern of m^6^A-associated lncRNA, model identification and group visualisation and risk modelling were carried out.

### Analysis of AL355574.1 in HCC

AL355574.1 in pan-cancer was obtained from the UCSC Xena database. RNA-seq data analysis of AL355574.1 in HCC and normal tissues was performed using TCGA-LIHC expression data. Kaplan-Meier analysis was used to calculate the association between AL355574.1 expression and OS in HCC patients. Relationship between AL355574.1 gene expression and clinicopathological features in HCC patients was analysed using Fisher's exact test. The relationship between AL355574.1 expression levels and HCC prognosis was calculated using univariate and multivariate Cox regression analysis was used to assess whether AL355574.1 could be an independent prognostic factor for survival in HCC patients. P<0.05 was considered statistically significant.

### GO, KEGG and GSEA enrichment analysis of AL355574.1 in HCC and comprehensive analysis of immune cell infiltration

To obtain the genes associated with AL355574.1 expression and to explore the biological functions and processes that AL355574.1 may be involved in HCC, Log FC>1 and P<0.05 were used as screening conditions. GO, KEGG and GSEA enrichment analyses were performed on these relevant genes. Enrichment analyses were carried out with "clusterprolifer", "Org.hs.eg.db", "ggplot" and "erichplot" in the R software. The correlation between the expression level of AL355574.1 and 22 immune cells was visualised and analysed using the "erichplot" package. P<0.05 was considered a statistically significant difference.

### Antibodies & reagents

The EdU cell proliferation detection assay was purchased from RiboBio Co. (Guangzhou, China). CCK-8 was purchased from Beyotime Biotech Co., Ltd (Shanghai, China). Anti-GAPDH (D16H11), anti-p-Akt (Ser473) and anti-p-mTOR (Ser2448) antibodies were purchased from Cell Signaling Technology (Beverly, MA, USA). E-cadherin (A20798), N-cadherin (A19083), MMP-9 (A0289) and MMP-2 (A6247) antibodies were all from ABclonal Biotechnology (Wuhan, China). Horseradish peroxidase-linked anti-mouse IgG and anti-rabbit IgG secondary antibodies were purchased from Cell Signaling Technology (Beverly, MA, USA).

### Clinical tissue samples

We collected 48 pairs of HCC tissues and adjacent tissues from the Department of Hepatobiliary Surgery, First Affiliated Hospital of Wannan Medical College (Wuhu, Anhui Province, China). All tissues were surgical biopsy samples and were evaluated by two pathologists in accordance with World Health Organization guidelines. All tissue samples from surgery were immediately stored under liquid nitrogen until use. Patients did not receive any local or systemic treatment before surgery. All patients signed an informed consent form before the use of these clinical data for research. All investigations and experiments were approved by the Clinical Research Ethics Committee of the First Affiliated Hospital of Wannan Medical College.

### RT-qPCR analysis

Total RNA, extracted from the cells and tissues using TRIzol reagent (Ambion; Thermo Fisher Scientific, Inc.), was reverse-transcribed into cDNA following the instructions provided with the cDNA synthesis kit (K1622; Fermentas; Thermo Fisher Scientific, Inc). Then the content and purity of RNA were determined using NanoDrop one (Thermo Fisher). GAPDH was used as control gene for lncRNA detection. Relative lncRNA expression level was determined by the 2^-ΔΔCt^ method.

### CCK-8 assay

The viability of Huh7 and HepG2 cells was determined using the CCK-8 assay. Briefly, Huh7 and HepG2 cells transfected with si-lncRNA or si-NC were seeded into 96-well cell culture plate at density of 1×10^4^/well. Following incubation for 24 h, 48 h, or 72 h, 10 µl/well of CCK-8 working fluid was added. The cells were then incubated for another 2 h. The absorbance value of each well was measured at 450 nm using a Multiskan™ GO plate reader. The experiment was repeated four times and the data were expressed as mean ± SD.

### EdU assay

The EdU assay was used to detect cell proliferation according to the manufacturer's protocol. Briefly, transfected Huh7 and HepG2 cells were seeded into 24-well plates. The cells were incubated with DMEM medium containing EdU for 2 h. After cell fixation (4% formaldehyde) and cell membrane permeabilisation (0.5% Triton X-100), the cells were stained with Apollo staining solution and Hoechst. Imaged using inverted fluorescence microscopy (Olympus, Tokyo, Japan). Image J version 1.52 software was used to analyze the results.

### Colony formation assay

Huh7 and HepG2 cells were seeded into 6-well plates and cultured in DMEM medium containing 10% FBS for 2 weeks. The medium was changed every 3 days. Cell colonies were stained with 0.1% crystal violet. After washing for 3 times with PBS, the colonies were photographed and the number of colonies was counted using Imaging J version 1.52 software.

### Transwell assay

Cell migration was determined by Transwell assay (Corning, NY, USA) with a pore size of 8.0 μm. Briefly, Huh7 and HepG2 cells transfected with AL355574.1 at the density of 2×10^4^ were suspended with 200 µl serum-free DMEM medium, which were added into the upper chamber. 600 µl DMEM medium containing 20% FBS was added to the lower chamber. The cells were then incubated for 24 h. After fixation with 4% formaldehyde for 10 min, the cells on the upper were swapped gently. The migrated cells were captured using an inverted microscope (Olympus, Tokyo, Japan) after staining with 0.1% crystal violet.

### Wound healing assay

Transfected Huh7 and HepG2 cells were seeded in 6-well cell culture plates, following monolayer fusion, cells were scraped with a sterile 200 µl pipette tip. After being washed with PBS, the cells were incubated in DMEM medium. Wound healing was photographed at 0 h, 24 h and 48 h, respectively using an inverted microscope (Olympus, Tokyo, Japan) and the results were analysed using Image J software version 1.52.

### Protein extraction and Western blot

Huh7 and HepG2 cells were washed once with pre-cooled PBS, and then RIPA cell lysis solution containing protease inhibitor (PMSF) (Beyotime, Shanghai, China) was added, and the cells were lysed on ice for 40 min, during which the cell culture plate was shaken frequently to ensure that the cells were sufficiently lysed. The cell lysates were collected and centrifuged for 10 min at 12,000 rpm at 4 ℃. Total protein was quantified using Nano Drop One (Thermo Fisher). Loading buffer was added to boil the protein for 10 min, an equal amount of protein was taken for SDS-PAGE, the electrophoresed protein was transferred to a nitrocellulose membrane, the membrane was blocked with skim milk powder for 1 h, washed 3 times with TBST, and then the indicated primary antibodies were added and incubated at 4 ℃ overnight. After washing with TBST for 3 times, the membrane was incubated with HRP-conjugated secondary antibody for another 1 h at room temperature. Finally, chemiluminescent solution and substrate were added, and protein bands were detected using a chemiluminescence imager (Clinx, Shanghai, China). Semi-quantitative detection of protein expression levels was performed using Image J 1.52 software.

### Statistical analysis

All analyses were performed using R-coding language, and data from different groups were compared using a t-test or Wilcoxon test. P<0.05 was considered statistically significant.

## Results

### Profiling of m^6^A RNA methylation regulators in HCC

**Figure 2:**
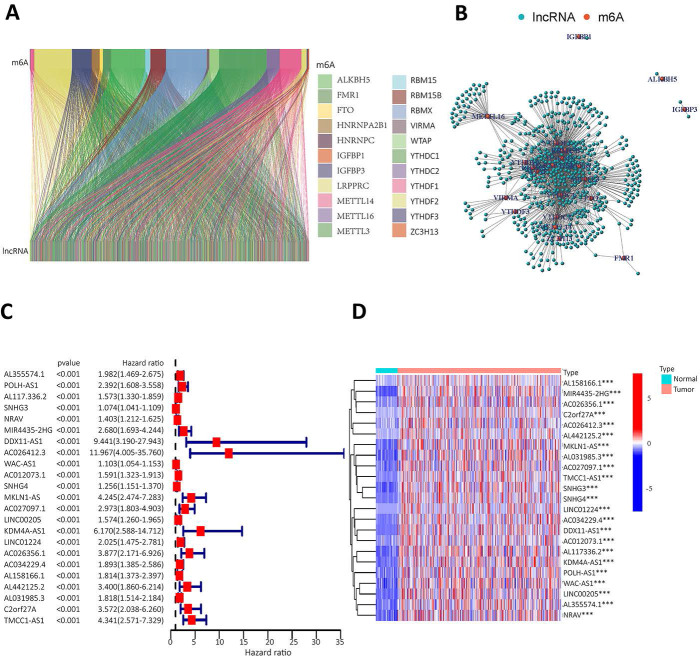
** To identify m^6^A gene-associated prognostic lncRNAs and their differential expression levels in HCC patients. A.** Expression Sankey diagram between lncRNA expression and m^6^A-related gene expression in HCC patients based on TCGA database; **B.** Correlation between m^6^A-associated gene expression and lncRNA expression in HCC patients using the TCGA database. **C.** The prognosis-related lncRNA expression data were analysed by univariate Cox regression and presented as a forest plot. Hazard ratios were calculated for the confidence intervals of correlated lncRNAs, and the red color represents high-risk lncRNAs; **D.** Differences in the expression of lncRNAs associated with the prognosis of m^6^A in HCC tissues and normal tissues were analysed on the basis of the TCGA database and presented as heatmaps.

Twenty-three gene expression data associated with m^6^A were extracted from the TCGA-LIHC transcriptome data, which was downloaded from the TCGA database. The mRNA genes were eliminated by differentiating mRNA from lncRNA, and then Sanji plots and network plots were drawn according to the correlation between m^6^A-related gene expression and lncRNA expression. Each colour in the Sanji plot represents an m^6^A gene. The different colour widths indicate the correlation between m^6^A and lncRNA, with wider colours indicating a closer relationship between m^6^A and lncRNA (Figure 2A). The red dots in the network diagram indicate m^6^A-related genes, and the surrounding green dots indicate lncRNAs associated with m^6^A genes (Figure 2B). Among them, FTO, METTL3, RBMX1 and YTHDC1 co-expressed lncRNAs more than other m^6^A-related genes. Univariate Cox regression analysis was then performed on the m^6^A-associated lncRNAs and the results are shown in the forest plot. The screened lncRNAs were considered to be m^6^A-associated prognostic lncRNAs, with a significance level of p<0.0001 (Figure 2C). Expression analyses of these prognostic lncRNAs were performed to observe their expression in HCC tissues and normal tissues. The results are shown in heat map (Figure 2D). The results showed that these prognosis-associated lncRNAs were highly expressed in HCC tissues.

### Analysing the role of m^6^A-related lncRNA and its effects on immune cell infiltration and tumor microenvironment

**Figure 3:**
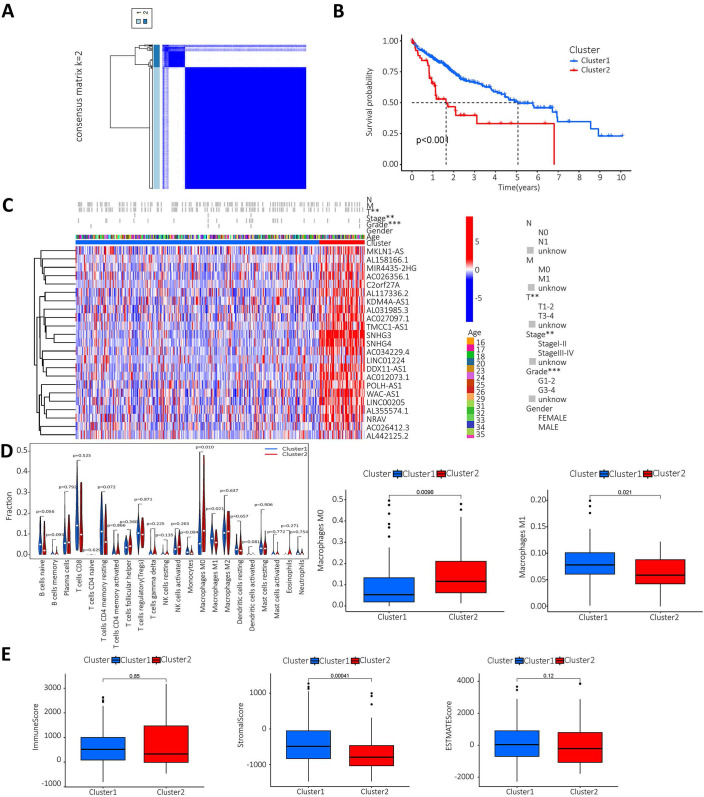
** Analysis of prognosis-related m^6^A-lncRNAs between survival, clinicopathological parameters, and immune infiltration in HCC patients. A.** According to the expression of lncRNAs, when K=2, there were least cross-mixing part between the two types and the CDF value was lowest, so we classified lncRNAs into two types: cluser 1 and cluster 2; **B.** Survival analysis according to the subtype group of different lncRNAs; **C.** Relationship between the difference in expression of prognostic lncRNAs in different lncRNA subtype groups and different clinicopathological parameters in HCC patients, red represents high expression clusters, blue represents low expression clusters, the horizontal axis represents samples, the vertical axis represents m^6^A-related prognostic lncRNAs; **D.** The differential analysis of the infiltration of immune cells in the different clusters is shown in the vioplot; **E.** Differential analysis of tumour microenvironment in different clusters was performed and results shown in vioplot.

The lncRNAs were categorised based on the expression of m^6^A prognostic lncRNAs. When K=2, the overlap was minimal and the CDF value was lowest. Then, the lncRNAs were divided into two groups, cluster1 and cluster2 (Figure 3A). We performed survival analysis on the two lncRNA clusters to evaluate the prognostic value of m^6^A-lncRNAs. The results showed that the survival time of cluster 2 was significantly shorter than that in cluster 1 (P<0.001) (Figure 3B). Subsequently, the correlation between the different lncRNAs in the two clusters and the clinicopathological characteristics of HCC patients was explored. The results indicated that high expression of these lncRNAs in cluster 2. These prognosis-related lncRNAs displayed a significant association with the T stage, pathological stage and histological grade. Figure 3C illustrated a heat map which depicted the correlation between the expression of prognosis-related lncRNAs and clinicopathological characteristics of HCC patients. To investigate and calculate the different immune cells infiltrated in the samples. Differences in immune cell infiltration in different clusters were analyzed and the differences were visualized using Vioplot. Furthermore, the infiltration level of each immune cell type in each different lncRNAs cluster was analyzed and boxplots drawn (Figure 3D). Macrophage M0 was highly clustered in cluster 2, whereas macrophage M1 was highly clustered in cluster 1 (P < 0.05). We also performed a differential analysis of the tumor microenvironment in different lncRNAs clusters of samples to determine the purity of different tumor cell types. The results were presented using a boxplot. As shown in Figure 3E, the StromalScore was higher in cluster 1, this indicates a lower purity of tumor cells and higher levels of immune-associated stromal cells in the tumor microenvironment.

### Construct a prognostic model for m^6^A-related lncRNAs based on the TCGA database

**Figure 4:**
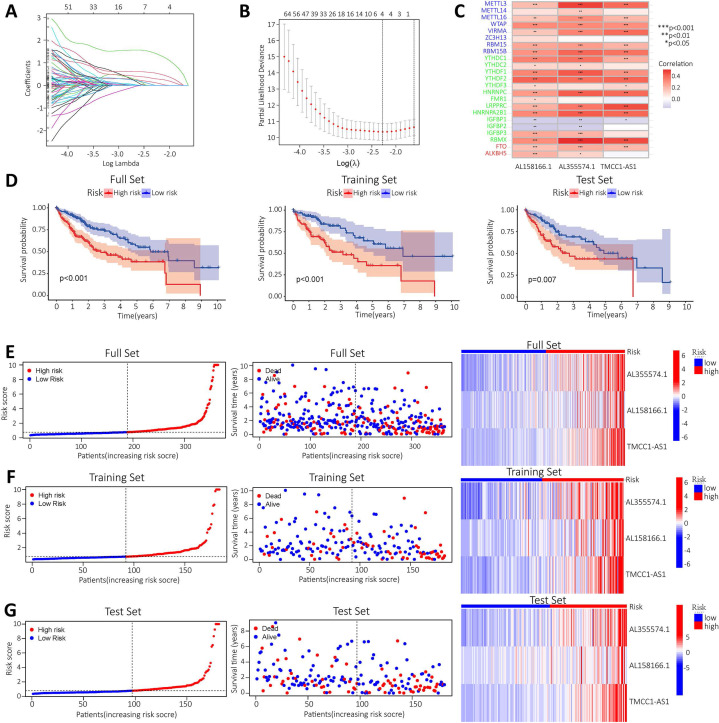
** Construction of a prognostic model for m^6^A-related lncRNAs based on the TCGA database. A-B.** Prognostic model was constructed via LASSO regression; **C.** Heat map of correlation of AL355574.1, AL158166.1 and TMCC1-AS1 with m^6^A gene; **D.** Kaplan-Meier curve analysis between the high-risk group and low-risk group was performed in the all-dataset, train data set and test data set; **E.** Patient risk scores and survivals with high and low-risk values in the all-dataset, train data set and test data set; Expression of m^6^A-associated lncRNA models for each patient presented in the cluster analysis heat map (AL35574.1, AL158166.1, and TMCC1-AS1); **F-G.** Correlation analysis results for the train and test dataset.

To construct a prognostic model of m^6^A-related lncRNA, HCC patients were divided into train and test group, the risk scores of the lncRNAs included in the model construction were calculated for each sample in the train group and the median of the risk scores was obtained. The train and test groups were classified as high-risk and low-risk groups according to the median risk value. Co-expression analysis and univariate Cox regression analysis yielded m^6^A-associated lncRNAs with prognostic value (Figure S1). To eliminate covariate collinearity and avoid overfitting of the prognostic model, lasso regression analysis was performed for the differentially expressed m^6^A-associated lncRNAs with prognostic value to calculate the hazard coefficient for each lncRNA. Four prognostic related lncRNAs were retained based on minimum partial likelihood deviance lncRNA (Figure 4A-B). Finally, a prognostic model for HCC patients was determined using m^6^A-lncRNA based on the expression values of three lncRNAs (AL158166.1, AL355574.1and TMCC1-AS1) (Figure 4C). The result shows the correlation between the modelled m^6^A gene and m^6^A-associated lncRNAs. The heat map showed a close relationship between AL158166.1 and YTHDF2; between AL355574.1 and METTL3, RBMX; and between TMCC1-AS1 and LRPPRC, RBMX.

HCC patients in the train and test group were categorised into high and low risk groups based on median risk scores. Based on survival curves, OS was lower in the high-risk subgroup than in the low-risk subgroup in the all-data group, train group and test group (Figure 4D). A risk curve was created to assessed the survival and risk of m^6^A-lncRNA (Figure 4E-G). Increases in risk score associated with increased number of deaths and increased proportion of high-risk patients. There were correlations between shorter survival, increased risk scores and increased mortality. The KM analysis showed the OS-rate of patients. It indicated that red represents dead patients, while blue represents those still alive. Patient survival decreases as the risk score increases. The heat map showed the expression levels of the three lncRNAs (AL158166.1, AL355574.1 and TMCC1-AS1), which were found to have higher expression in the high-risk group compared to the low-risk group. three lncRNAs were identified as having a high association with m^6^A prognosis. In order to assess the prognostic ability of the established model, a standard formula was used to calculate the risk score for each patient in both the train and test groups. The figure shows the pattern plots for risk categorisation, survival status and survival time in the all-data group, train group and test group (Figure 4E shows the all-data group, while Figure 4F and Figure 4G show the train and test groups, respectively).

### Independent prognostic value of the lncRNA prognostic risk model

**Figure 5:**
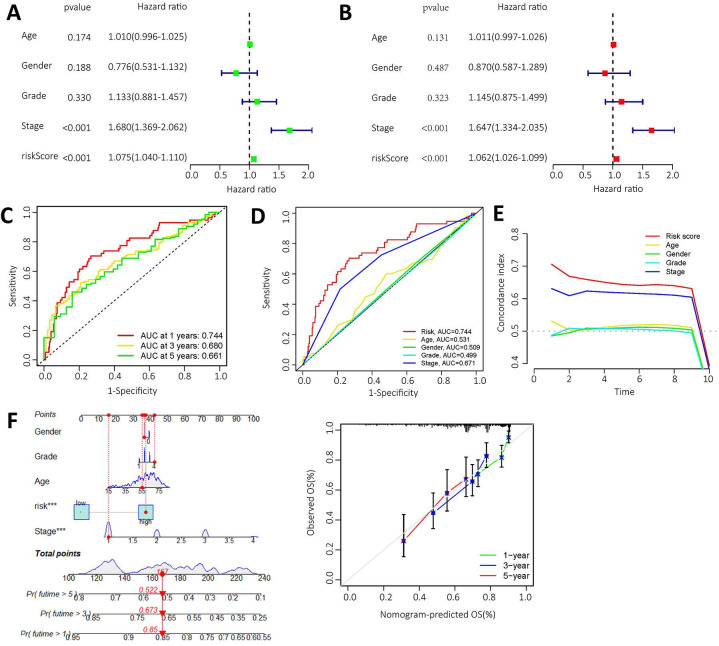
** Independent prognostic value of risk model for m^6^A-lncRNA. A.** Forest plot of univariate Cox regression suggest that the pathological stage and risk score of HCC patients are relevant prognostic values; **B.** Forest plot of multivariate Cox regression showing pathological stage and risk score as independent prognostic factors in HCC patients; **C.** The ROC curves show the sensitivity of the risk scores in predicting the survival of patients with HCC (sensitivity of patient survival at 1, 3 and 5 years). The ROC curves represent the reliability of the risk models described above;** D.** ROC curve showing the sensitivity of risk model, age, gender, pathological stage, and histological grade; **E.** The c-index (exponential curve) shows that the risk score is the most sensitive factor; **F.** The likelihood of 1-year, 3-year and 5-year OS predicted by the nomogram, and the likelihood of 1-year, 3-year and 5-year OS predicted by the calibration curve of the nomogram.

High expression levels of AL355574.1, AL158166.1 and TMCC1-AS1 were found in the constructed models, where AL355574.1 (HR=3.370366, 95%cl (1.863747-6.094909)), AL158166.1 (HR=4.022717, 95%cl (2.448033-6.610308)) and TMCC1-AS1 (HR=25.65064, 95%cl (8.616655-76.35855)) had higher hazard ratios. We performed independent prognostic analyses to assess whether our model was independent of other clinical prognostic factors that affects patient prognosis. Hazard ratios for different indicators were calculated in univariate and multivariate Cox regression analyses. The results showed that both the pathological stage of the patients and the model risk score could serve as independent prognostic risk factors (P<0.05) (Figure 5A-B). The time-dependent ROC curves showed that the AUC values at 1, 3 and 5 years were 0.661, 0.680 and 0.744 (Figure 5C).

Thus, ROC curves confirmed the prognostic value of the risk score. This was higher than the AUC values for age, gender, pathological stage and histological grade. This result suggests that the risk model we have constructed is more sensitive than the other indicators (Figure 5D). C-index curve shows that the risk scores were more closely related to the prognosis of patients with HCC (Figure 5E), The risk scores had higher indices compared to other clinical indicators. We constructed a nomogram including risk levels and clinical risk characteristics to predict OS at 1, 3 and 5 years. The data of Figure 5F revealed that there was strong concordance between the observed and predicted rates.

### PCA further validated the grouping ability of the m^6^A-associated lncRNA model

**Figure 6:**
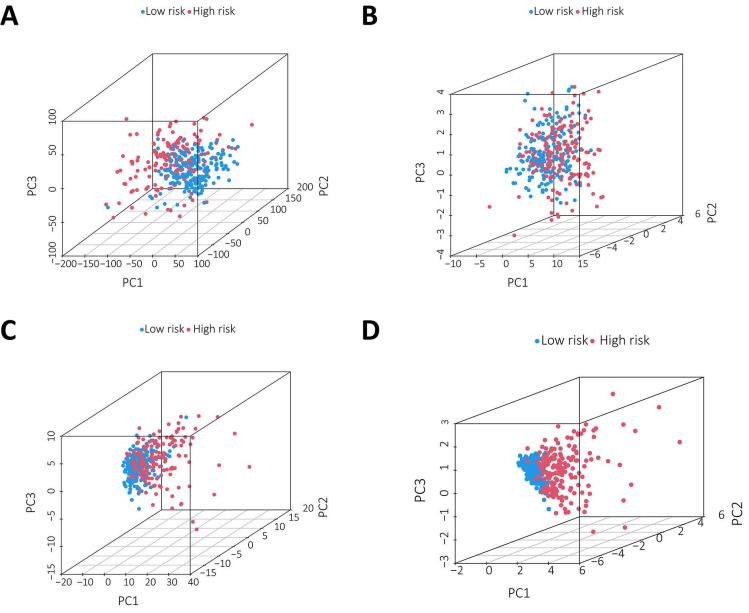
Principal component analysis between the two groups based on** A.** total gene expression profile;** B.** 23 m^6^A genes; **C.** m^6^A-associated lncRNAs and** D.** 3 m^6^A-associated lncRNA profiles based on TCGA database.

PCA was conducted to test the difference between the low-risk and high-risk groups based on the entire gene expression profiles, 23 m^6^A genes, 3 m^6^A-related lncRNAs, and risk model classified by the expression profiles of the 3 m^6^A-related lncRNAs (Figure 6A-D). As shown in Figure 6A-C, the distributions of the high-risk and low-risk groups were relatively dispersed. However, there was a difference in the distribution between the two groups (Figure 6D). These results suggest that the prognostic signature can distinguish between the low-risk and high-risk groups.

### Enrichment Analysis and Mutation Analysis

**Figure 7:**
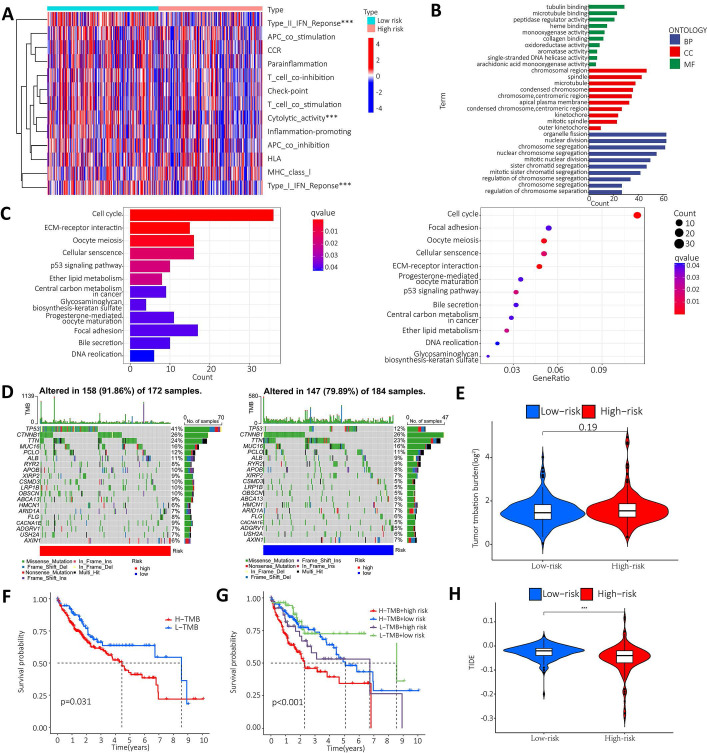
** Enrichment analysis and mutation analysis. A.** The correlation between the high-risk and low-risk groups in terms of immune function; **B.** GO results show that model molecules are associated with the biological processes of Tubulin binding/Microtubule Binding and peptidase regulator activity; **C.** KEGG results show that model molecules are associated with biological processes such as cell cycle, ECM-receptor integration and P53 signaling pathway; **D.** Differences in mutation types of different genes in HCC patients in the high-risk and low-risk groups; **E.** Violin plot showen that the difference between high-risk and low risk groups in TMB; **F.** Difference in survival time between TMB patients in the high-risk group and the low-risk group; **G.** The prognosis in different groups; **H.** Violin plot showed the difference between high and low-risk groups in TIDE.

To further clarify the functional differences of specific molecules between low-risk and high-risk groups, enrichment levels and activity of various immune pathways and functions were examined. The results showed that there were significant differences between the two groups in the expression of most immune indices. Immune functions such as type I IFN response, type II IFN response and cytolytic activity showed a significant correlation between the high and low risk groups (Figure 7A). GO and KEGG enrichment analyses were performed to further explore the potential biological mechanisms of the m^6^A-associated lncRNA model. GO results from the MF group showed that the model molecules were associated with Tubulin binding, microtubule binding and peptidase regulator activity of biological processes. The CC results showed that the model molecules were associated with the biological processes of chromosome region, spindle and microtubule. The BP results showed that the model molecules were associated with the biological processes of organelle fission, nuclear division and chromosome segregation. The KEGG results showed that the model molecules were associated with biological processes such as the cell cycle, ECM receptor integration and the P53 signaling pathway (Figure 7B-C). Summary analysis of the mutation data using the R package Maftools, which stratifies mutations according to mutation effects and predictors, revealed the top 20 driver genes with the highest frequencies between the two subgroups. The highest proportion of TP53 mutations was found in the high-risk group (approximately 41%), according to waterfall plots obtained from the mutation analysis. The highest proportion of TP53 mutations was found in missense mutations, followed by frameshift del mutations. The highest proportion of CTNNB1 mutations was found in the low-risk group (approximately 26%). The highest proportion of CTNNB1 mutations was found in the missense mutation group (Figure 7D). The TMB score was then calculated from the TCGA-LIHC mutation data. The results showed no significant difference between the high-risk and low-risk groups (Figure 7E). The KM survival analysis of TMB in samples from the high risk and low risk groups showed a lower survival rate in the high risk group with a high TMB (Figure 7F). Further analysis showed that the high mutation and high-risk groups had a poor prognosis, the low mutation and low risk groups had a better prognosis, and the low mutation and high-risk groups had a poor prognosis than the high mutation and low risk groups (Figure 7G). Tumor immune dysfunction and rejection (TIDE) suggested the efficacy of tumor immune escape and immune checkpoint blockade therapy in HCC. The findings showed that the low-risk group was more likely to respond to immunotherapy than the high-risk group. The result suggests that the m^6^A-related lncRNA model can be used as a predictor of TIDE, and these findings are consistent with our previous results (Figure 7H).

### Upregulation of lncRNA AL355574.1 expression in HCC

**Figure 8:**
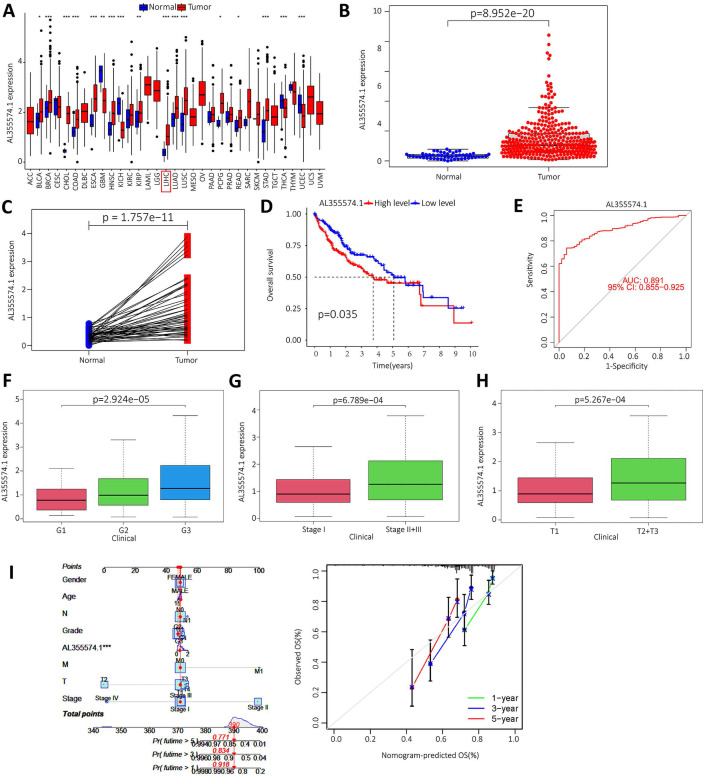
** Expression level and prognosis of AL355574.1 in HCC samples. A.** The expression of AL355574.1 in different tumor tissues; **B-C.** The expression levels of AL355574.1 were elevated in HCC based on the TCGA database; **D.** Based on the TCGA database, the overall survival time of HCC patients with high AL355574.1 expression was significantly shorter than that of patients with low AL355574.1 expression;** E.** For the TCGA database, elevated AL355574.1 expression in HCC subjects with working characteristic analysis (AUC=0.891) had diagnostic value;** F-H.** AL355574.1 expression was positively correlated with Histological grade, Pathological stage and T stage; **I.** Likelihood of OS at 1, 3, and 5 years in HCC patients predicted by prediction plots. Likelihood of calibration plots of 1-year, 3-year, and 5-year OS prediction column plots.

We downloaded the LIHC data from the TCGA database, and the raw data are shown in Table S1. The expression of AL355574.1 in different cancer types was analysed using the UCSC-Xena database. It was found that AL355574.1 expression was increased in the majority of tumors (Figure 8A). In addition, analysis of TCGA-LIHC database revealed that AL355574.1 expression was significantly higher in tumor tissues than that in normal tissues (p=8.952e-20) (Figure 8B). Pair-wise comparison between cancer and adjacent normal tissues obtained the same conclusion as Figure 8B (p=1.757e-11) (Figure 8C). Kaplan-Meier survival curves showed that patients with high AL355574.1 expression had shorter survival times than those with low AL355574.1 expression (Figure 8D). AL355574.1 has certain diagnostic value in HCC patients (ROC=0.891) and can affect the prognosis of HCC patients (Figure 8E). HCC patients were divided into high and low AL355574.1 gene expression groups according to the median expression level of AL355574.1 gene. Statistically significant differences in pathological stage, histological grade, T stage and risk factors were found between the two groups, suggesting that differences in AL355574.1 gene expression levels may affect the clinicopathological progression and prognostic survival of patients (Table S2). The expression of AL355574.1 mRNA was observed in each group according to the clinicopathological characteristics of the HCC patients using Wilcoxon signed rank test and logistic regression. The expression level of AL355574.1 mRNA was higher in patients with high histological grade (Figure 8F), pathological stage (Figure 8G) and T stage (Figure 8H) than that in patients with low grade. The relationship between AL355574.1 gene expression and clinicopathological characteristics of HCC patients was analysed using univariate logistic regression. AL355574.1 gene expression was significantly associated with T stage, histological grade, pathological stage and risk factors (Table S3). The above results suggest that AL355574.1 gene has the potential to be an indicator of HCC stage. Univariate Cox regression showed that pathological stage, T stage, M stage and AL355574.1 gene expression were risk factors for HCC. Multivariate Cox regression analysis showed that AL355574.1 gene expression was an independent prognostic factor for OS (Table S4). The expression of AL355574.1 and other clinical parameters were constructed to build predictive models to predict the OS of patients at 1, 3 and 5 years. Based on the column line plots, the expression level of AL355574.1 showed significant predictive power compared to the clinical factors. The correlation legends showed that there was good agreement between the observed and predicted rates of OS at 1 year, 3 years and 5 years (Figure 8I).

### GO, KEGG and GSEA enrichment score of AL355574.1, as well as immune infiltration and potential drug analysis

**Figure 9:**
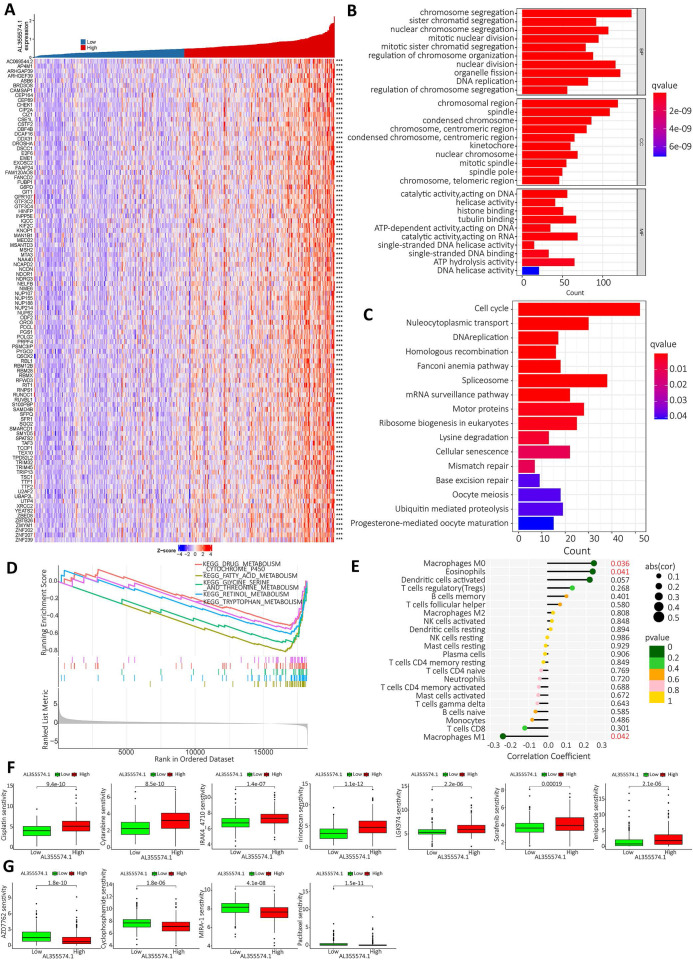
** AL355574.1 GO, KEGG enrichment analysis and GSEA enrichment score as well as immune infiltration. A.** The top 100 genes associated with AL355574.1 expression. **B.** The biological functional pathways associated with AL355574.1 were identified using GO enrichment analysis;** C.** KEGG enrichment analysis, and GSEA **D**; **E.** Relationship between AL355574.1 expression levels and the relative abundance of 22 immune cell types; **F-G.** Sensitivity of drugs between patients with high and low AL355574.1 expression.

Based on the expression of AL355574.1, the top 100 genes with significant differences were screened for visual analysis (Figure 9A). GO enrichment analysis showed that AL355574.1-related genes in HCC were mainly enriched in chromosome segregation, sister chromatid segregation and nuclear chromosome segregation (Figure 9B). KEGG enrichment showed that AL355574.1-related genes were mainly enriched in cell cycle, nucleocytoplasmic transport and DNA replication (Figure 9C). GSEA enrichment showed that AL355574.1-related genes were mainly enriched in drug metabolism Cytochrome p450 and Fatty acid metabolism (Figure 9D). To comprehensively investigate the role of AL355574.1 in HCC, we analyzed the association between AL355574.1 expression level and infiltrating immune cell subpopulations. We found that AL355574.1 gene expression was positively correlated with macrophage M0 and eosinophils, while negatively correlated macrophage M1 (Figure 9E). The screening results revealed that 11 potential therapeutic agents showed different degrees of sensitivity in the high and low AL355574.1 susceptible population. For cisplatin, cytarabine, IRAK4-4710, irinotelan, LGK974, sorafenib and teniposide, the IC50 of the low AL355574.1 expression group was lower than that of the high expression group. This indicated that patients in the low AL355574.1 expression group were more sensitive to these drugs. For AZD7762, Cyclophosphamide, MIRA-1 and Paclitaxel, the IC50 AL355574.1 low expression arm was higher than that of the high expression group, indicating that patients in the AL355574.1 high expression group were more sensitive to these drugs (Figure 9F-G). AL355574.1 was found to be associated with ferroptosis and disulfidptosis in gastric cancer^20^, ^21^. This prompted us to further analyse the association between AL355574.1 and ferroptosis, cuproptosis and disulfidptosis in HCC patients. Figure S2-S4 suggested that there was a correlation between AL355574.1 and some of the genes related to ferroptosis, cuproptosis and disulfidptosis in HCC patients. In the future, we will focus on the role and molecular mechanism of AL355574.1 in ferroptosis, cuproptosis and disulfidptosis in HCC.

### High-expression of AL355574.1 in HCC tissues and its effect on Huh7 and HepG2 cell proliferation and migration

**Figure 10:**
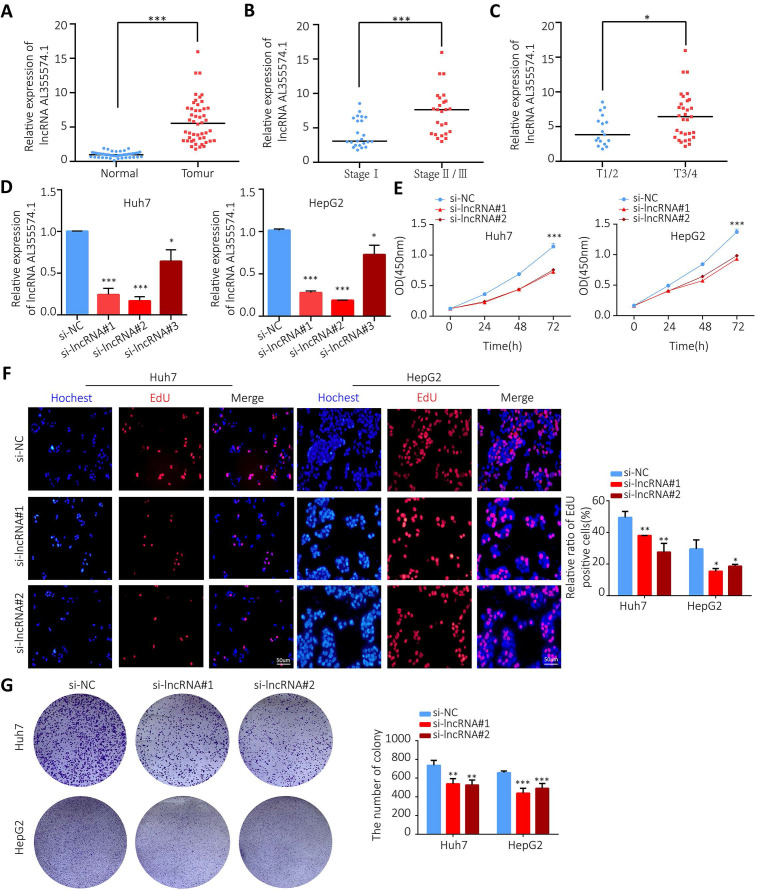
** The expression of AL355574.1 in HCC tissues and the effect of AL355574.1 on HCC cells proliferation. A.** AL355574.1 was highly expressed in HCC tissues. **B-C.** The expression of AL355574.1 in the different pathological stage and T-stages of clinical patients. Huh7 and HepG2 cells were transfected with si-AL355574.1 and negative control siRNA (si-NC) for the indicated times, RT-qPCR was used to verify the knockdown efficiency** (D)**; CCK-8 assay was used to detect the cell viability **(E)**; EdU and colony formation assays were used to measure the cell proliferation **(F-G).** Scale bar=50 μm, data were shown as mean±SD.*P < 0.05, **P < 0.01 and ***P < 0.001.

AL355574.1 mRNA expression levels in 48 pairs of tumors and adjacent normal tissues were examined by RT-qPCR. Our data found that AL355574.1 expression was higher in HCC tissues than that in adjacent normal tissues (Figure 10A). The expression of AL355574.1 correlated with the pathological stage and T-stages of clinical patients, and with increasing clinicopathological grade, the expression level of AL355574.1 was increased (Figure 10B-C). The correlation between AL355574.1 expression and clinical parameters of HCC patients was analysed using Fisher's exact test.

As shown in Table 1, the results indicated that the pathological stage, the tumor size (cm) and the OS were statistically different between the two groups. To further investigate the function of AL355574.1 in HCC, we transfected siRNA AL355574.1 and si-NC into the Huh7 and HepG2 cells, respectively. Our data showed that siRNA AL355574.1#1 and #2 had higher knockdown efficiencies (Figure 10D). CCK-8 results showed that knockdown of AL355574.1 in Huh7 and HepG2 cells obviously inhibited the cell activity (Figure 10E). EdU assays showed that AL355574.1 knockdown significantly inhibited Huh7 and HepG2 cell proliferation (Figure 10F).

**Figure 11:**
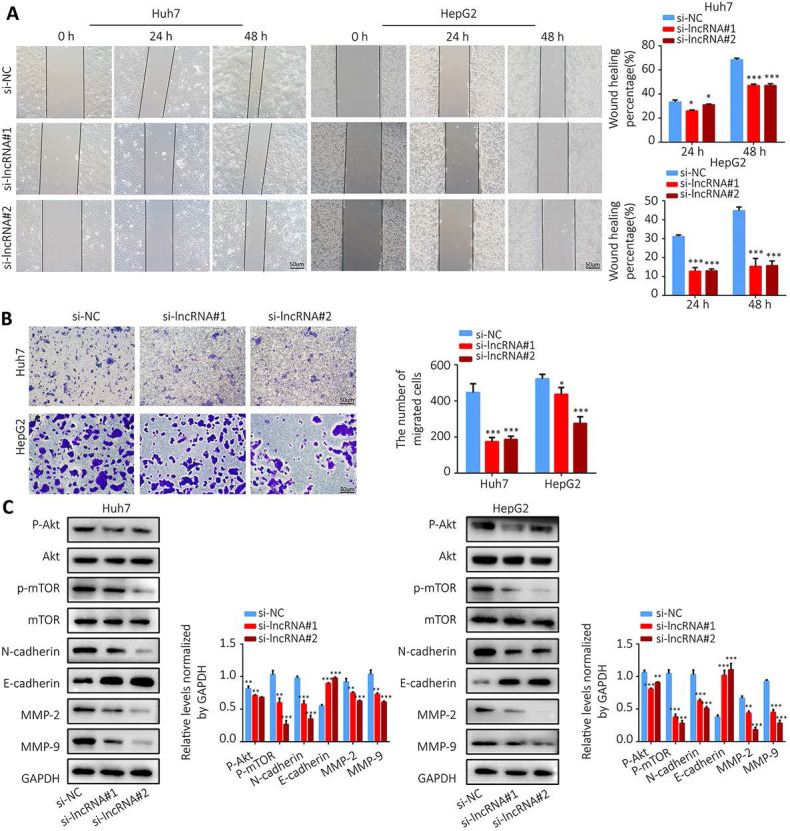
** The effect of AL355574.1 on migration of HCC cells.** Huh7 and HepG2 cells were transfected with si-AL355574.1 and negative control siRNA, wound healing assay **(A)** and transwell assay **(B)** were used to investigate the effects of AL355574.1 on cell migration, respectively, the expression levels of MMP-2, MMP-9, E-cadherin, N-cadherin and the phosphorylation of Akt and mTOR all were measured using Western blotting** (C).** Scale bar=50 μm, data were shown as mean±SD.*P < 0.05, **P < 0.01 and ***P < 0.001.

Colony formation assay showed that knockdown of AL355574.1 expression led to smaller and fewer colonies (Figure 10G). The above results suggest that AL355574.1 serves as a pro-proliferation agent for HCC cells *in vitro*. Subsequently, we examined the effect of AL355574.1 on HCC cell migration. The wound healing assay showed that knockdown of AL355574.1 led to a decrease in the rate of cell migration (Figure 11A). In the Transwell assay, the number of migrated cells was decreased in AL355574.1 knockdown group compared to the si-NC group (Figure 11B). MMP-2 and MMP-9, two important matrix metalloproteinases, have a significant impact on the invasion and metastasis of cancer cells^22^. The epithelial-mesenchymal transition (EMT) involved in the early stages of metastasis is characterized by the upregulation of N-cadherin and downregulation of E-cadherin^23^. The Akt/mTOR signalling pathway has been reported to regulate cell proliferation and frequently activated in human cancer^24^, ^25^. Once activated, Akt/mTOR regulates the function of many downstream proteins that may be involved in HCC proliferation and migration. Western blotting results showed that AL355574.1 knockdown significantly inhibited the phosphorylation level of Akt/mTOR, decreased the expression of MMP-2, MMP-9 and N-cadherin, but increased the expression of E-cadherin (Figure 11C).

## Discussion

HCC is one of the most common cancers worldwide, with arising mortality rate which is contrary to the trend of decreasing mortality observed in other cancers^26^. Therefore, it is essential to conduct regular screenings of high-risk individuals^27^. Early detection of the cancer increases the chances of successful surgical resection, with a five-year survival rate of more than 20%. The current standard for the early detection of HCC is the use of ultrasound along with serum AFP levels and cross-sectional imaging for liver nodules that are larger than 1 cm^28^. Further research has identified potential biomarkers in serum/plasma proteins and circulating DNA/RNA markers that offer promising diagnostic ability in HCC surveillance^29^. The methylation modification of m^6^A is one of the most common RNA modifications with implications for normal biological function and disease progression, and is strongly associated with gene expression, thus highlighting the potential role of m^6^A in carcinogenesis^30^. LncRNA, a major type of non-coding RNA, similar to mRNA^31^, the m^6^A modifications regulate their gene expression^32^. M^6^A-associated lncRNAs have been shown to determine tumor prognosis, marking them of potential use in the diagnosis and therapy of HCC. For example, m^6^A-mediated upregulation of LINC00958 increases adipogenesis and could be a nanotherapeutic target for HCC^33^. METTL16 promotes HCC progression by downregulating RAB11B-AS1 in an m^6^A-dependent manner^34^. Lipopolysaccharide facilitates immune escape of HCC cells via m^6^A modification of lncRNA MIR155HG, leading to the upregulate of PD-L1 expression^35^. Therefore, the identification and analysis of m^6^A-lncRNAs in HCC is crucial as it could provide a direction for research and possible therapeutic targets for HCC treatment.

In this study, to investigate the role and significance of m^6^A-associated lncRNAs in HCC, we extracted the expression data of 23 m^6^A-associated genes and distinguished between mRNAs and lncRNAs. Co-expression analysis was performed to determine the expression correlation between the 23 m^6^A-associated genes and lncRNAs, leading to the construction of a m^6^A-lncRNA gene expression network map. This indicated that some lncRNAs were associated with m^6^A-related genes in HCC. Therefore, we speculated that m^6^A-related lncRNAs might regulate the biological functions of HCC. Prognostically relevant lncRNAs were identified based on differential P values, confidence intervals and risk ratios. We screened m^6^A-related lncRNAs that displayed a close association with HCC prognosis via univariate Cox analysis. We found that there were 23 m^6^A-associated lncRNAs that were prognostic in HCC, and with high expression in HCC tissues, and they may play an important role in cancer progression.

Due to the role of m^6^A-lncRNA modification in cancer progression, we further investigated the role of the m^6^A-lncRNAs in HCC. We performed survival analyses according to the different lncRNA subtype groups to assess the prognostic value of m^6^A-lncRNAs. To determine the role of immune cell infiltration and TME in HCC, the different immune cell infiltrations of different lncRNA subtype groups were also examined and calculated. The differences analysis of immune cell infiltration showed that macrophage M0 immune cells highly infiltrated in cluster 2 tumor tissue, whereas macrophage M1 immune cells highly infiltrated in cluster 1 tumor tissue. The higher the immune score indicates the lower the tumor purity and better the prognosis. Then, to further explore the relationship between m^6^A-associated lncRNAs and the immune microenvironment, we constructed and validated an internal HCC prognostic model using lasso regression. Three m^6^A-associated lncRNAs (AL355574.1, AL158166.1 and TMCC1-AS1) were identified. It has been shown that gastric cancer patients with low expression of AL355574.1 had short survival rate or time and poor prognosis^36^. The AL158166.1 was found to be associated with immune infiltration in HCC and laryngeal squamous cell carcinoma^37^. TMCC1-AS1 was identified as a predicator of a poor prognosis and an accelerated epithelial-mesenchymal transition in HCC^38^. HCC patients were divided into high-risk and low-risk subcategories according to median risk. Patients in the high-risk cohort have a significantly poorer prognosis than those in the low-risk cohort. The trend was consistent in both the test and train groups. Thus, the above data suggested that the m^6^A lncRNA-associated prognostic model can predict the prognosis of HCC.

In addition, the accuracy of our model in predicting the survival of patients with this disease was quite high. Increasing risk scores were associated with increased deaths and an increased proportion of high-risk patients. We also used Cox regression and ROC analysis to validate the accuracy of the model, these results confirm that our m^6^A-associated lncRNA model can accurately predict the survival of HCC patients. Immunotherapy plays an important role in the treatment of malignant tumors, the study of potential mutated genes can aid in the diagnosis and rational selection of treatment. In this study, there was a significant difference in immune function between the high-risk and low-risk groups. In addition, the mutation rates of the TP53 and CTNNB1 genes were high in both the high-risk group and the low-risk group. CTNNB1 mutations were found in about 18-40% of HCC patients. The Wnt/β-catenin protein pathway activated by CTNNB1 mutations plays a key role in regulating liver metabolism. The TP53 gene mutations are common in tumors and affect T-cell recruitment and activity, result in immune escape. These results suggest that m^6^A-related lncRNA gene mutations are closely associated with immune activity in HCC patients.

Taken together, above study showed that m^6^A-lncRNA may be a suitable clinical model for the prediction of HCC outcome. Gene expression and survival analyses showed that AL355574.1 gene expression levels were higher in HCC patients, and patients with high AL355574.1 expression had shorter survival time. These further confirmed that AL355574.1 may be a pro-carcinogenic gene in HCC. Multivariate Cox regression analysis showed that AL355574.1 gene expression was an independent prognostic factor associated with overall survival of HCC patients. Evaluation the relationship between AL355574.1 expression levels and immune cells showed that there was a correlation between the expression levels of AL355574.1 and macrophages M0, eosinophils and macrophages M1. In addition, analysis showed that the expression level of AL355574.1 correlated with some genes related with ferroptosis, cuproptosis and disulfidptosis.

We validated the possible biological function and underlying molecular mechanism of AL355574.1 on HCC at the cellular level. EMT has been reported to be a transformative process required for the local and distant migration of HCC. MMPs play an important role in regeneration, programmed death and angiogenesis of HCC cells^39^. Akt/PKB (protein kinase B) is a serine-threonine kinase involved in a variety of important cellular pathways, including survival, proliferation, invasion, apoptosis and angiogenesis^40^. Our data showed that knockdown of AL355574.1 significantly inhibited the proliferation and migration ability of Huh7 and HepG2 cells, suppressed the expression of MMP-2, MMP-9, N-cadherin and Akt/mTOR phosphorylation, but promoted the expression of E-cadherin. These results suggest that AL355574.1 promotes proliferation and migration of HCC cells via Akt/mTOR signaling pathway, MMPs expression and EMT. The bioinformatics analyses and in vitro experimental results both suggested that AL355574.1 may be a potential biomolecular marker and immunotherapeutic target for HCC.

## Conclusion

We confirmed the prognostic value of m^6^A-associated lncRNAs by analysing gene expression profiles and clinical data of HCC samples in the TCGA database. The role of m^6^A-lncRNA in HCC was confirmed by immune cell infiltration and prognostic models. Subsequently, it was found that the AL355574.1 was highly correlated with the OS of patients with HCC. Differential expression analysis and correlation analysis further clarified the prognostic value of AL355574.1. We further identified the involved signaling pathways by GSEA. AL355574.1 was positively correlated with the immune cell macrophages M0 and eosinophils, and negatively correlated with the immune cell macrophages M1. Our study advances the understanding of m^6^A-associated lncRNAs and provides novel therapeutic targets and prognostic biomarkers for HCC.

## Supplementary Material

Supplementary figures and tables.

## Figures and Tables

**Table 1:** ** Correlation between AL355574.1 and clinical parameters.** Fisher's exact test was used for statistical analysis.

Parameter	Number	AL355574.1 expression		*P* Value
		Low	High	
**Gender**				0.734
Female	11	6	5	
Male	37	18	19	
**Age (years)**				0.77
<=60	29	14	15	
>60	19	10	9	
**Tumor size (cm)**				**0.002**
<=5.5	25	18	7	
>5.5	23	6	17	
**Pathologic Stage**				**0.045**
Stage I	23	15	8	
Stage II/III	25	9	16	
**T-stage**				0.135
T1/T2	17	11	6	
T3/T4	31	13	18	
**N-stage**				1
N0	48	24	24	
N1	0	0	0	
**M-stage**				1
M0	46	23	23	
M1	2	1	1	
**OS event**				**0.018**
Alive	30	19	11	
Dead	18	5	13	

## Abbreviations

GO: Geneontology
KEGG: Kyoto encyclopedia of genes and genomes
HCC: hepatocellular carcinoma
LIHC: Liver Hepatocellular Carcinoma
m^6^A: N^6^-methyladenosine
TCGA: The Cancer Genome Atlas
lncRNA: long non-coding RNAs lncRNAs
LASSO: least absolute shrinkage and selection operator
TMB: Tumor Mutational Burden
ceRNA: competitive endogenous RNA
OS: overall survival
HR: hazard ratio
