# Supplementary material for: Analysis of m6A-related lncRNAs for prognostic and immunotherapeutic response in hepatocellular carcinoma
Source: J Cancer. 2024 Feb 17;15(7):2045–65. doi: 10.7150/jca.92128 (PMC10905389; doi:10.7150/jca.92128)
Supplement: Supplementary file 1 — Supplementary figures and tables. [file jcav15p2045s1.pdf]

Supplements

Figure S1. m6A-associated lncRNAs with prognostic value were identified by co-expression analysis and univariate cox regression analysis.

	pvalue	Hazard ratio
RNASEH1-AS1	0.026	2.275(1.102-4.696)
MAPKAPK5-AS1	0.015	1.597(1.097-2.327)
ATP1A1-AS1	0.049	3.180(1.003-10.085)
AL355574.1	<0.001	3.370(1.864-6.095)
AC099850.1	0.005	3.171(1.406-7.151)
AL662791.1	0.019	2.241(1.144-4.390)
AL513320.1	0.047	2.164(1.009-4.640)
POLH-AS1	0.028	2.553(1.109-5.874)
Z97989.1	0.037	4.117(1.091-15.540)
AC010973.2	0.037	3.836(1.083-13.590)
AL357079.1	0.034	2.698(1.080-6.737)
AC022007.1	0.012	2.581(1.230-5.415)
AC004943.2	0.007	4.132(1.461-11.683)
AL161729.4	0.017	3.304(1.238-8.814)
AL050341.2	0.028	1.546(1.049-2.280)
AL117336.2	0.003	2.553(1.365-4.775)
CHROMR	0.019	3.476(1.228-9.837)
AC244033.2	0.042	1.738(1.021-2.958)
AC018645.3	0.046	1.650(1.010-2.697)
SNHG16	0.004	1.997(1.247-3.199)
LINC02035	0.011	2.141(1.191-3.850)
AL358472.4	0.013	2.149(1.178-3.922)
AP001469.2	0.028	3.607(1.149-11.326)
AC083855.2	0.003	1.753(1.215-2.529)
SNHG7	0.016	1.477(1.075-2.028)
LYRM4-AS1	0.006	3.133(1.379-7.119)
LINC01836	0.045	1.370(1.007-1.863)
AL606489.1	0.025	1.565(1.059-2.312)
AP003469.2	0.003	1.705(1.206-2.410)
GPRC5D-AS1	0.025	2.002(1.091-3.673)
ARIH2OS	0.009	2.234(1.223-4.080)
AL122035.1	0.010	3.773(1.373-10.373)
AC113189.3	0.001	3.147(1.562-6.341)
AL109627.1	0.004	2.227(1.283-3.867)
SNHG3	0.001	1.563(1.187-2.058)
RHPN1-AS1	0.042	1.885(1.025-3.467)
ZEB1-AS1	0.002	2.363(1.354-4.123)
AC005332.6	0.019	1.750(1.097-2.792)
SNHG1	0.036	1.485(1.027-2.149)
NRAV	<0.001	2.669(1.660-4.292)
ZNF337-AS1	0.043	3.397(1.042-11.079)
LINC02709	<0.001	2.850(1.582-5.132)
SCAMP1-AS1	0.024	2.252(1.111-4.568)
AC125437.1	0.045	2.317(1.020-5.265)
Z99572.1	0.015	0.192(0.051-0.723)
AC005332.5	0.019	1.907(1.111-3.274)
AC006504.7	0.017	1.779(1.106-2.860)
MIR4435-2HG	0.002	3.612(1.574-8.288)
AL158212.3	0.047	2.308(1.011-5.269)
AC009061.2	0.011	4.527(1.422-14.409)
AP003390.1	<0.001	3.713(1.723-8.003)
SBF2-AS1	0.034	2.057(1.057-4.003)
AC091057.1	0.008	2.755(1.302-5.827)
WAC-AS1	0.011	1.790(1.145-2.799)
HMGN3-AS1	0.003	3.350(1.529-7.338)
AC107959.3	0.004	1.681(1.183-2.388)
ARRDC1-AS1	0.048	1.633(1.004-2.657)
AC012073.1	<0.001	2.817(1.623-4.888)
SNHG4	<0.001	2.019(1.372-2.971)
LINC00653	0.048	2.376(1.008-5.602)
AC083799.1	0.008	1.802(1.166-2.784)
SNHG20	0.013	2.580(1.220-5.457)
AC108479.1	0.008	2.333(1.253-4.344)
MKLN1-AS	<0.001	5.906(2.640-13.212)
NIFK-AS1	0.004	2.666(1.372-5.182)
AC115619.1	<0.001	0.756(0.645-0.886)
JMJD1C-AS1	0.008	4.805(1.501-15.381)
AC027097.1	0.007	3.483(1.401-8.658)
LINC00205	0.004	2.267(1.302-3.947)
BACE1-AS	0.004	4.964(1.651-14.929)
AC092614.1	0.047	2.944(1.016-8.535)
LINC00654	0.020	2.970(1.187-7.431)
CLCA4-AS1	0.028	2.594(1.107-6.079)
LINC01011	0.001	2.986(1.528-5.835)
AC112220.2	0.023	2.586(1.137-5.878)
KDM4A-AS1	0.049	3.533(1.005-12.426)
AC021851.1	0.017	4.043(1.288-12.691)
AC004803.1	<0.001	7.753(2.307-26.060)
AC004816.1	0.005	2.066(1.252-3.410)
SLC25A21-AS1	0.014	2.250(1.178-4.295)
AC016747.1	0.014	1.910(1.142-3.195)
AL499602.1	0.011	1.384(1.078-1.777)
AL354892.2	0.048	1.507(1.003-2.266)
USP46-DT	0.010	2.158(1.203-3.869)
AL078644.1	0.017	3.206(1.229-8.359)
AL133215.2	0.012	2.802(1.260-6.235)
GIHCG	0.014	1.855(1.133-3.039)
AC145207.5	0.013	2.875(1.254-6.594)
NRSN2-AS1	0.037	2.481(1.058-5.816)
HCG15	0.002	3.982(1.647-9.632)
THUMPD3-AS1	0.020	2.242(1.134-4.430)
EIF2AK3-DT	0.045	2.456(1.021-5.909)
AC005670.3	0.002	7.577(2.045-28.069)
LINC01224	0.003	2.394(1.340-4.277)
SNHG12	0.012	1.809(1.142-2.867)
AP001469.3	0.034	2.247(1.064-4.747)
AC025176.1	<0.001	2.786(1.561-4.972)
AC026401.3	0.003	1.516(1.148-2.002)
AC010761.1	0.039	2.245(1.042-4.837)
AC026356.1	<0.001	5.904(2.399-14.531)
ITPR1-DT	0.034	3.773(1.104-12.898)
AL592148.3	0.032	3.899(1.122-13.545)
WARS2-AS1	0.047	2.036(1.010-4.104)
AC034229.4	0.027	2.334(1.099-4.959)
AC010186.3	0.018	2.346(1.157-4.758)
AL031670.1	0.019	3.560(1.232-10.292)
AL158166.1	<0.001	4.023(2.448-6.610)
AL442125.2	0.010	4.270(1.421-12.837)
ZBTB11-AS1	0.037	2.308(1.050-5.074)
LINC02820	0.021	1.848(1.098-3.112)
RNF216P1	0.007	2.337(1.256-4.346)
TBC1D8-AS1	0.006	4.305(1.522-12.176)
AL031985.3	<0.001	2.822(1.651-4.824)
AL590705.3	0.002	2.636(1.410-4.929)
AL359504.1	0.019	2.026(1.123-3.658)
AC002398.1	0.002	3.559(1.566-8.090)
AC116351.1	0.030	1.458(1.038-2.047)
CENATAC-DT	0.028	2.004(1.077-3.730)
AL592295.4	0.026	1.985(1.084-3.636)
TMCC1-AS1	<0.001	25.651(8.617-76.359)
AC016065.1	0.022	2.459(1.136-5.326)
FAM111A-DT	0.002	2.698(1.422-5.120)

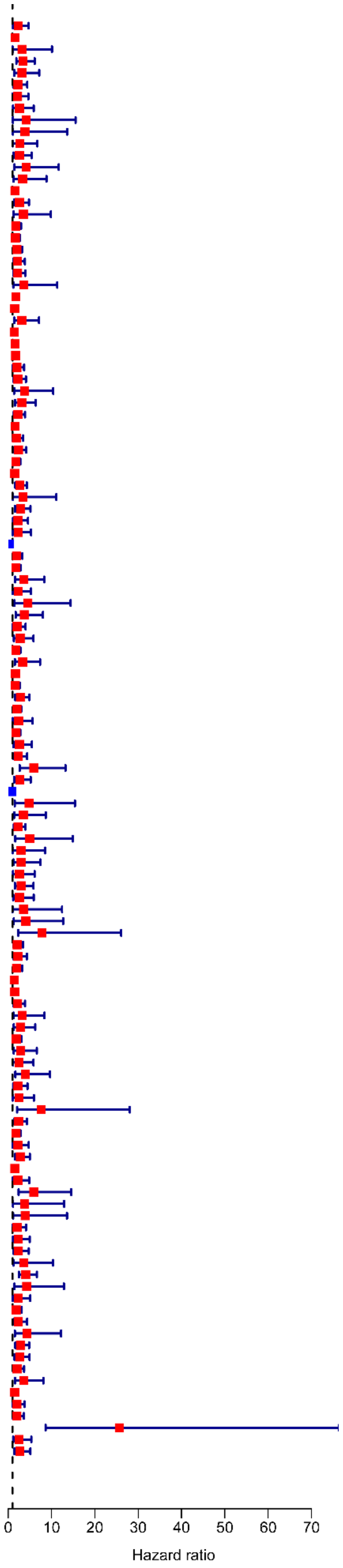

Figure S2. The association of lncRNA AL355574.1 with ferroptosis related genes in HCC tissues.

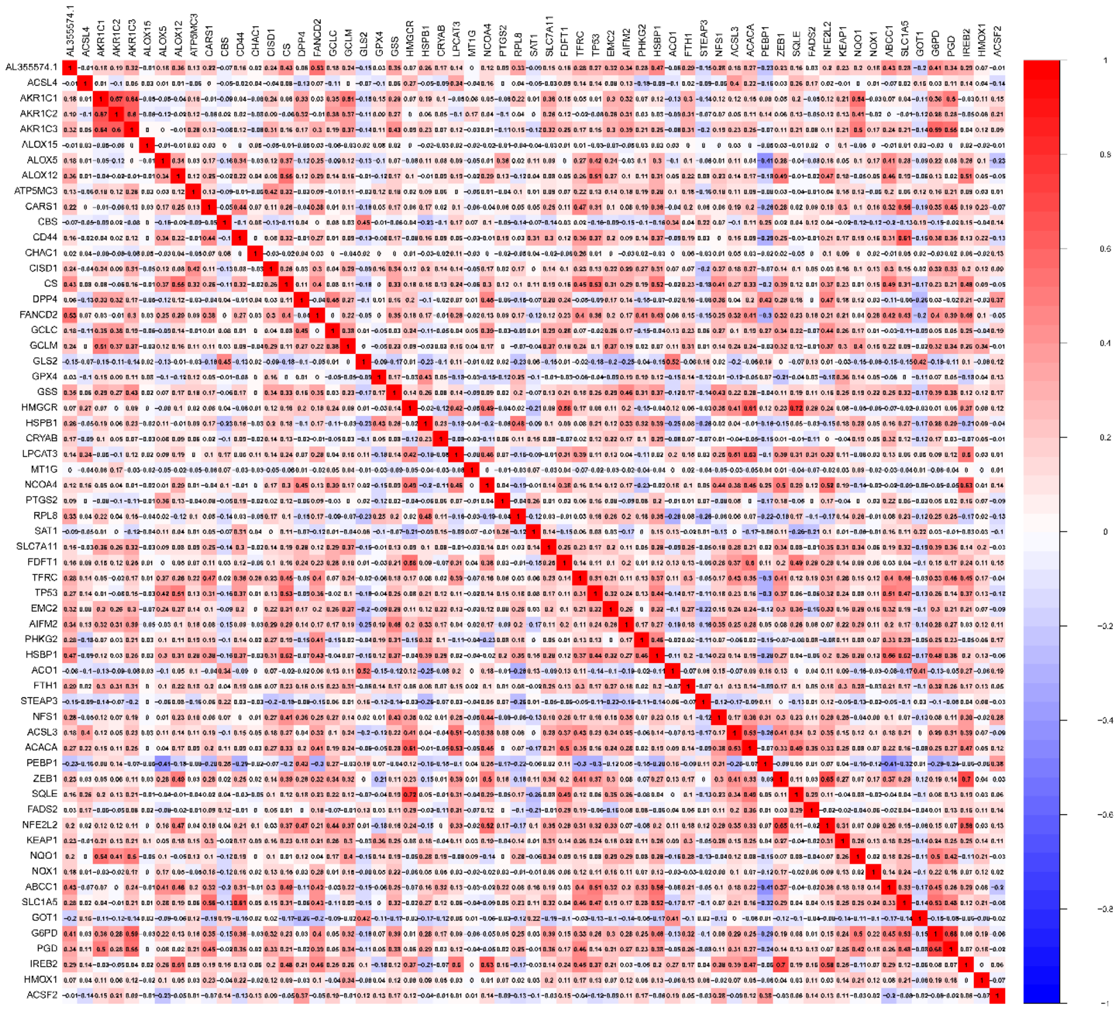

Figure S3. The association of lncRNA AL355574.1 with cuproptosis related genes in HCC tissues.

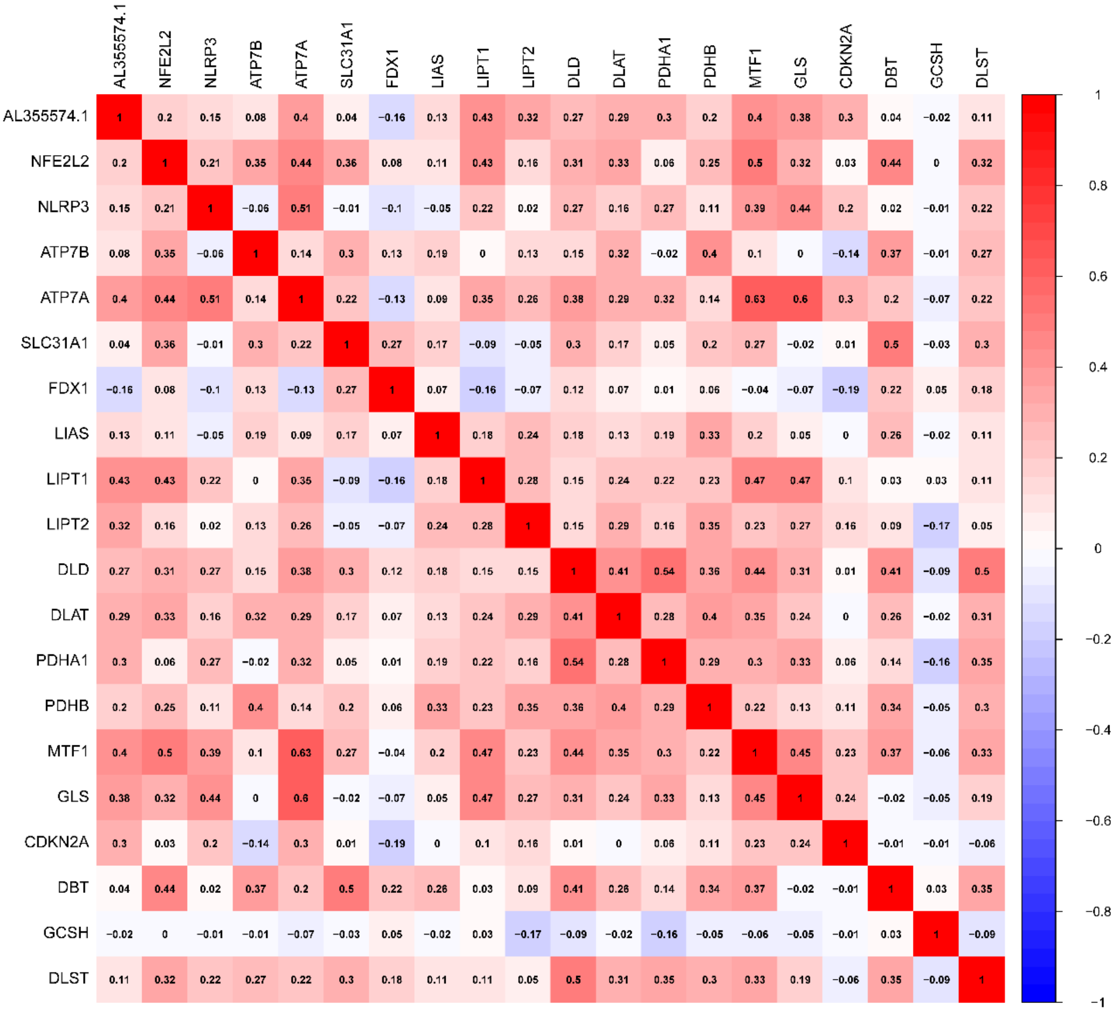

Figure S4. The association of lncRNA AL355574.1 with disulfidptosis related genes in HCC tissues.

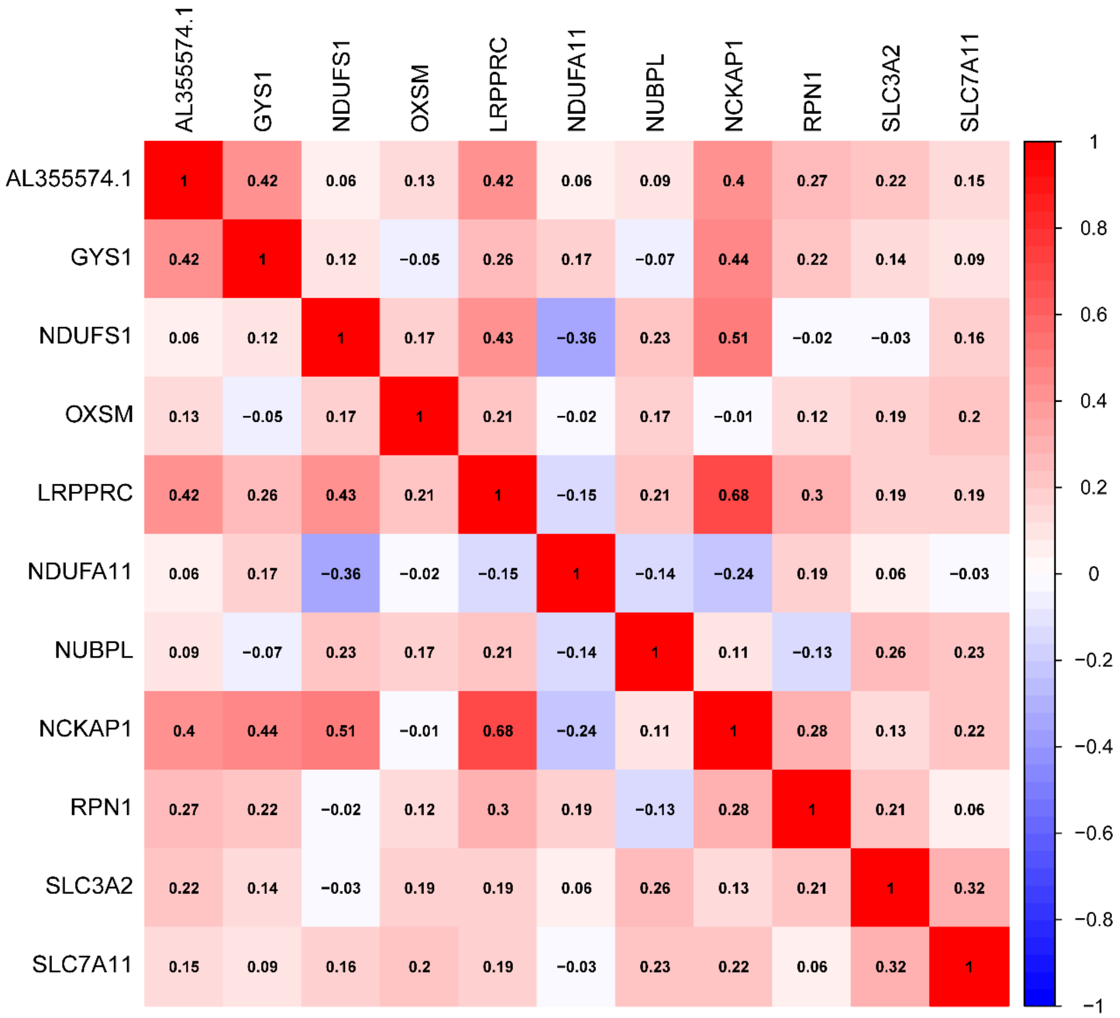

Table S1. The raw data about LIHC in TCGA database

ID	Status	Type	OS	Age	Vital status	Gender	Race	Residual tumor	Histological grade	M stage	N stage	T stage	Pathologic stage	AFP
TCGA-2V-A95S	Tumor	LIHC	unknow	unknow	Alive	MALE	ASIAN	R0	G3	MX	NX	T2	Stage II	10793
TCGA-2Y-A9GS	Tumor	LIHC	724	58	Dead	MALE	WHITE	R0	G2	MX	NX	T2	unknow	74
TCGA-2Y-A9GT	Tumor	LIHC	1624	51	Dead	MALE	WHITE	R0	G2	MX	NX	T1	Stage I	17
TCGA-2Y-A9GU	Tumor	LIHC	1939	55	Alive	FEMALE	WHITE	R0	G2	MX	NX	T1	Stage I	304
TCGA-2Y-A9GV	Tumor	LIHC	2532	54	Dead	FEMALE	WHITE	R0	G1	MX	NX	T1	Stage I	6
TCGA-2Y-A9GW	Tumor	LIHC	1271	64	Dead	MALE	WHITE	R0	G2	MX	N0	T1	Stage I	2
TCGA-2Y-A9GX	Tumor	LIHC	2442	68	Alive	MALE	WHITE	R1	G2	MX	NX	T1	Stage I	1
TCGA-2Y-A9GY	Tumor	LIHC	757	64	Dead	FEMALE	WHITE	R0	G3	MX	NX	T2	Stage II	27600
TCGA-2Y-A9GZ	Tumor	LIHC	848	82	Dead	FEMALE	WHITE	R0	G2	MX	NX	T2	Stage II	7
TCGA-2Y-A9H0	Tumor	LIHC	3675	49	Alive	MALE	WHITE	R0	G1	M0	N0	T3	Stage IIIA	7598
TCGA-2Y-A9H1	Tumor	LIHC	1229	58	Dead	MALE	WHITE	R0	G2	MX	NX	T1	Stage I	3
TCGA-2Y-A9H2	Tumor	LIHC	1731	64	Alive	FEMALE	WHITE	R0	G3	MX	N0	T1	Stage I	2
TCGA-2Y-A9H3	Tumor	LIHC	1516	45	Alive	MALE	WHITE	R0	G1	MX	NX	T2	Stage II	5640

TCGA-2Y-A9H4	Tumor	LIHC	1452	68	Alive	MALE	BLACK OR AFRICAN AMERICAN	R0	G2	MX	N0	T1	Stage I	11
TCGA-2Y-A9H5	Tumor	LIHC	555	59	Dead	FEMALE	WHITE	R0	G3	MX	N0	T1	Stage I	11700
TCGA-2Y-A9H6	Tumor	LIHC	357	68	Alive	FEMALE	WHITE	R0	G2	MX	NX	T1	Stage I	114
TCGA-2Y-A9H7	Tumor	LIHC	1168	81	Alive	FEMALE	WHITE	R0	G2	MX	N0	T1	Stage I	6
TCGA-2Y-A9H8	Tumor	LIHC	633	85	Dead	FEMALE	WHITE	R0	G2	MX	NX	T1	unknow	234000
TCGA-2Y-A9H9	Tumor	LIHC	697	70	Alive	MALE	WHITE	R0	G2	MX	N0	T1	Stage I	12
TCGA-2Y-A9HA	Tumor	LIHC	36	70	Dead	MALE	WHITE	R0	G2	MX	NX	T2	Stage II	114
TCGA-2Y-A9HB	Tumor	LIHC	260	66	Alive	MALE	unknow	R0	G2	MX	NX	T1	Stage I	21
TCGA-3K-AAZ8	Tumor	LIHC	396	65	Alive	MALE	BLACK OR AFRICAN AMERICAN	RX	G1	MX	NX	T3b	Stage IIIB	unknow
TCGA-4R-AA8I	Tumor	LIHC	262	66	Alive	MALE	WHITE	R0	G2	MX	NX	T2	Stage II	5
TCGA-5C-A9VG	Tumor	LIHC	328	58	Alive	MALE	WHITE	R0	G2	M0	N0	T2	Stage II	unknow
TCGA-5C-A9VH	Tumor	LIHC	322	70	Alive	MALE	WHITE	R0	G2	M0	N0	T1	Stage I	unknow
TCGA-5C-AAPD	Tumor	LIHC	20	61	Alive	MALE	ASIAN	R0	G1	M0	N0	T2	Stage II	unknow
TCGA-5R-AA1C	Tumor	LIHC	364	57	Alive	MALE	WHITE	R0	G2	M0	N0	T2	Stage II	2
TCGA-	Tumor	LIHC	337	17	Alive	FEMALE	WHITE	R0	G3	M0	N0	T3a	Stage IIIA	5

5R-AA1D														
TCGA-5R-AAAM	Tumor	LIHC	46	65	Dead	FEMALE	WHITE	R0	G2	M0	N0	T2	Stage II	32
TCGA-BC-4072	Tumor	LIHC	1490	74	Dead	FEMALE	WHITE	R1	G3	M0	N0	T3	Stage IIIA	unknow
TCGA-BC-4073	Tumor	LIHC	352	73	Alive	MALE	WHITE	R1	G3	MX	N0	T3	Stage IIIA	unknow
TCGA-BC-A10Q	Tumor	LIHC	1135	72	Dead	FEMALE	WHITE	R1	unknow	MX	NX	T2	unknow	unknow
TCGA-BC-A10R	Tumor	LIHC	308	66	Dead	FEMALE	WHITE	R0	G2	MX	NX	T3	unknow	27
TCGA-BC-A10S	Tumor	LIHC	1423	81	Dead	MALE	WHITE	R0	G1	MX	NX	T3	unknow	unknow
TCGA-BC-A10T	Tumor	LIHC	837	76	Dead	MALE	WHITE	R1	G1	MX	NX	T4	unknow	unknow
TCGA-BC-A10U	Tumor	LIHC	837	69	Dead	MALE	WHITE	R1	G2	MX	NX	T2	unknow	38
TCGA-BC-A10W	Tumor	LIHC	91	50	Dead	MALE	ASIAN	R1	G3	MX	NX	T4	unknow	79
TCGA-BC-A10X	Tumor	LIHC	770	52	Dead	FEMALE	WHITE	R0	G2	MX	N0	T3a	Stage IIIA	264
TCGA-BC-A10Y	Tumor	LIHC	711	76	Dead	MALE	WHITE	R0	G3	MX	NX	T4	unknow	10575
TCGA-BC-A10Z	Tumor	LIHC	34	62	Dead	FEMALE	WHITE	R0	G2	MX	N0	T1	Stage I	779
TCGA-BC-A110	Tumor	LIHC	2116	51	Dead	FEMALE	BLACK OR AFRICAN AMERICAN	R0	G1	MX	NX	T1	unknow	unknow
TCGA-BC-A112	Tumor	LIHC	153	80	Dead	MALE	WHITE	R0	G2	MX	NX	T3	unknow	233

TCGA-BC-A216	Tumor	LIHC	1351	62	Alive	FEMALE	WHITE	R0	G2	M0	NX	T3	Stage IIIA	831
TCGA-BC-A217	Tumor	LIHC	421	75	Alive	FEMALE	WHITE	R0	G3	M0	NX	T2	Stage II	28
TCGA-BC-A3KF	Tumor	LIHC	8	66	Alive	FEMALE	WHITE	R0	G2	M0	NX	T1	Stage I	unknow
TCGA-BC-A3KG	Tumor	LIHC	498	68	Alive	FEMALE	WHITE	R0	G3	M0	N0	T2	Stage II	618
TCGA-BC-A5W4	Tumor	LIHC	547	69	Alive	MALE	WHITE	R1	G3	M0	NX	T3a	Stage IIIA	unknow
TCGA-BC-A69H	Tumor	LIHC	444	64	Alive	MALE	WHITE	R0	G3	M0	NX	T2	Stage II	103900
TCGA-BC-A69I	Tumor	LIHC	387	69	Alive	MALE	WHITE	R0	G1	M0	N0	T1	Stage I	2
TCGA-BC-A8YO	Tumor	LIHC	562	66	Alive	FEMALE	WHITE	R0	G3	M0	N0	T4	Stage IIIC	unknow
TCGA-BD-A2L6	Tumor	LIHC	1363	69	Alive	MALE	WHITE	R0	G2	MX	NX	T2	unknow	53
TCGA-BD-A3EP	Tumor	LIHC	409	75	Alive	FEMALE	BLACK OR AFRICAN AMERICAN	R0	G2	M0	N0	T1	Stage I	17
TCGA-BD-A3ER	Tumor	LIHC	1115	62	Alive	MALE	WHITE	R0	G2	MX	NX	T2	Stage II	5
TCGA-BW-A5NO	Tumor	LIHC	20	50	Alive	MALE	BLACK OR AFRICAN AMERICAN	RX	G2	MX	NX	T3a	Stage IIIA	unknow
TCGA-BW-A5NP	Tumor	LIHC	unknow	26	Alive	FEMALE	WHITE	RX	G3	M1	N0	T2	Stage IV	143684
TCGA-BW-A5NQ	Tumor	LIHC	unknow	63	Alive	MALE	WHITE	RX	G3	MX	NX	T1	Stage I	unknow
TCGA-	Tumor	LIHC	129	48	Alive	MALE	ASIAN	RX	G2	M0	N0	T2	Stage II	unknow

CC-5258														
TCGA-CC-5259	Tumor	LIHC	250	60	Alive	FEMALE	ASIAN	R0	G2	M0	N0	T4	Stage IIIC	unknow
TCGA-CC-5260	Tumor	LIHC	87	61	Alive	FEMALE	ASIAN	RX	G1	M0	N0	T4	Stage IIIC	unknow
TCGA-CC-5261	Tumor	LIHC	97	44	Alive	MALE	ASIAN	RX	G2	M0	N0	T2	Stage II	unknow
TCGA-CC-5262	Tumor	LIHC	103	67	Alive	MALE	ASIAN	unknow	G1	M0	N0	T4	Stage IIIC	unknow
TCGA-CC-5263	Tumor	LIHC	129	35	Dead	MALE	ASIAN	RX	G1	M0	N0	T3	Stage IIIA	unknow
TCGA-CC-5264	Tumor	LIHC	102	71	Alive	MALE	ASIAN	RX	G2	M0	N0	T3	Stage IIIA	unknow
TCGA-CC-A123	Tumor	LIHC	219	24	Alive	FEMALE	ASIAN	RX	G1	M0	N0	T3	Stage IIIA	unknow
TCGA-CC-A1HT	Tumor	LIHC	101	50	Alive	MALE	ASIAN	unknow	G3	M0	N0	T3	Stage IIIA	unknow
TCGA-CC-A3M9	Tumor	LIHC	300	45	Alive	MALE	ASIAN	R0	G3	M0	N0	T3	Stage IIIA	unknow
TCGA-CC-A3MA	Tumor	LIHC	303	61	Alive	MALE	ASIAN	R0	G2	M0	N0	T3	Stage IIIA	unknow
TCGA-CC-A3MB	Tumor	LIHC	315	36	Alive	MALE	ASIAN	R0	G1	M0	N0	T3	Stage IIIA	unknow
TCGA-CC-A3MC	Tumor	LIHC	363	54	Alive	MALE	ASIAN	R0	G2	M0	N0	T3	Stage IIIA	unknow
TCGA-CC-A5UC	Tumor	LIHC	347	63	Alive	MALE	ASIAN	R0	G3	M0	N0	T3	Stage IIIA	unknow
TCGA-CC-A5UD	Tumor	LIHC	304	45	Alive	MALE	ASIAN	R0	G2	M0	N0	T3	Stage IIIA	unknow

TCGA-CC-A5UE	Tumor	LIHC	272	48	Alive	MALE	ASIAN	R0	G2	M0	N0	T4	Stage IIIB	unknow
TCGA-CC-A7IE	Tumor	LIHC	217	57	Alive	MALE	ASIAN	R0	G2	M0	N0	T3	Stage IIIA	unknow
TCGA-CC-A7IF	Tumor	LIHC	649	59	Alive	MALE	ASIAN	R0	G1	M0	N0	T3	Stage IIIA	unknow
TCGA-CC-A7IG	Tumor	LIHC	299	47	Alive	MALE	ASIAN	R0	G2	M0	N0	T2	Stage II	unknow
TCGA-CC-A7IH	Tumor	LIHC	365	58	Alive	MALE	ASIAN	R0	G1	M0	N0	T3	Stage IIIA	unknow
TCGA-CC-A7II	Tumor	LIHC	399	54	Alive	MALE	ASIAN	R0	G3	M0	N0	T3	Stage IIIA	unknow
TCGA-CC-A7IJ	Tumor	LIHC	382	56	Alive	MALE	ASIAN	R0	G3	M0	N0	T2	Stage II	unknow
TCGA-CC-A7IK	Tumor	LIHC	262	59	Alive	MALE	ASIAN	R0	G3	M0	N0	T3	Stage IIIA	unknow
TCGA-CC-A7IL	Tumor	LIHC	278	61	Alive	MALE	ASIAN	R0	G1	M0	N0	T3	Stage IIIA	unknow
TCGA-CC-A8HS	Tumor	LIHC	300	18	Alive	MALE	ASIAN	R0	G1	M0	N1	T3	Stage IIIC	unknow
TCGA-CC-A8HT	Tumor	LIHC	140	74	Alive	MALE	ASIAN	R0	G2	M0	N0	T3	Stage IIIA	unknow
TCGA-CC-A8HU	Tumor	LIHC	344	39	Alive	FEMALE	ASIAN	R0	G3	M0	N0	T3	Stage IIIA	unknow
TCGA-CC-A8HV	Tumor	LIHC	279	51	Alive	FEMALE	ASIAN	R0	G2	M0	N0	T2	Stage II	unknow
TCGA-CC-A9FS	Tumor	LIHC	211	55	Alive	MALE	ASIAN	R0	G2	M0	N0	T2	Stage II	unknow
TCGA-	Tumor	LIHC	unknow	52	Alive	FEMALE	ASIAN	R0	G2	M0	N0	T3a	Stage IIIA	unknow

CC-A9FU														
TCGA-CC-A9FV	Tumor	LIHC	unknow	57	Alive	MALE	ASIAN	R0	G2	M0	N0	T3	Stage IIIA	unknow
TCGA-CC-A9FW	Tumor	LIHC	248	68	Alive	MALE	ASIAN	R0	G2	M0	N0	T3	Stage IIIA	unknow
TCGA-DD-A113	Tumor	LIHC	2425	55	Alive	FEMALE	WHITE	R0	G3	M0	N0	T2	Stage II	133
TCGA-DD-A114	Tumor	LIHC	1149	42	Dead	MALE	BLACK OR AFRICAN AMERICAN	R0	G3	M0	unknow	T2	Stage II	21
TCGA-DD-A115	Tumor	LIHC	2542	53	Dead	MALE	WHITE	R0	G2	M0	N0	T3	Stage IIIA	10
TCGA-DD-A116	Tumor	LIHC	1622	68	Dead	MALE	ASIAN	R0	G3	M0	N0	T3	Stage IIIA	24
TCGA-DD-A118	Tumor	LIHC	3437	77	Alive	FEMALE	WHITE	R0	G2	M0	N0	T2	Stage II	85150
TCGA-DD-A119	Tumor	LIHC	223	40	Dead	MALE	ASIAN	R2	G3	M1	N0	T3a	Stage IV	1836
TCGA-DD-A11A	Tumor	LIHC	79	67	Alive	MALE	BLACK OR AFRICAN AMERICAN	R0	G3	M0	N0	T1	Stage I	223
TCGA-DD-A11B	Tumor	LIHC	14	73	Dead	MALE	WHITE	RX	G2	M0	N0	T1	Stage I	15
TCGA-DD-A11C	Tumor	LIHC	662	69	Alive	MALE	WHITE	RX	G3	M0	N0	T1	Stage I	11
TCGA-DD-A11D	Tumor	LIHC	1560	57	Dead	FEMALE	WHITE	R0	G2	M0	N0	T1	Stage I	8
TCGA-DD-A1E9	Tumor	LIHC	2759	70	Dead	MALE	WHITE	R0	G2	M0	N0	T1	Stage I	202
TCGA-DD-A1EA	Tumor	LIHC	2415	68	Alive	MALE	ASIAN	R0	G2	M0	N0	T2	Stage II	3

TCGA-DD-A1EB	Tumor	LIHC	2017	72	Alive	FEMALE	unknow	R0	G2	M0	N0	T1	Stage I	7
TCGA-DD-A1EC	Tumor	LIHC	602	20	Alive	FEMALE	WHITE	R0	G3	M0	N0	T1	Stage I	2
TCGA-DD-A1ED	Tumor	LIHC	2301	68	Alive	MALE	WHITE	R0	G1	M0	N0	T1	Stage I	3
TCGA-DD-A1EE	Tumor	LIHC	349	73	Alive	MALE	WHITE	R0	G3	M0	N0	T3	Stage IIIA	3
TCGA-DD-A1EF	Tumor	LIHC	394	57	Dead	FEMALE	WHITE	R0	G3	M0	N0	T1	Stage I	16211
TCGA-DD-A1EG	Tumor	LIHC	1372	76	Dead	MALE	WHITE	R0	G3	M0	N0	T1	Stage I	16
TCGA-DD-A1EH	Tumor	LIHC	1495	23	Alive	MALE	WHITE	R0	G3	M0	N0	T3	Stage III	94340
TCGA-DD-A1EI	Tumor	LIHC	183	46	Alive	MALE	ASIAN	R0	G2	M0	N0	T1	Stage I	24
TCGA-DD-A1EJ	Tumor	LIHC	1005	71	Alive	FEMALE	WHITE	R0	G2	M0	N1	T1	Stage IIIC	18840
TCGA-DD-A1EK	Tumor	LIHC	558	64	Dead	FEMALE	WHITE	R0	G2	M1	N0	T4	Stage IVB	19
TCGA-DD-A1EL	Tumor	LIHC	415	23	Dead	MALE	BLACK OR AFRICAN AMERICAN	R0	G3	M0	N0	T2	Stage II	5
TCGA-DD-A39V	Tumor	LIHC	643	77	Dead	MALE	WHITE	R0	G3	M0	NX	T2	Stage II	unknow
TCGA-DD-A39W	Tumor	LIHC	827	29	Dead	FEMALE	WHITE	R0	G2	M0	N0	T3	Stage III	unknow
TCGA-DD-A39X	Tumor	LIHC	1694	78	Dead	FEMALE	WHITE	R0	G2	M0	NX	T1	Stage I	10
TCGA-	Tumor	LIHC	171	67	Dead	MALE	ASIAN	R0	G3	M0	NX	T1	Stage I	5600

DD-A39Y													
TCGA-DD-A39Z	Tumor	LIHC	601	43	Dead	FEMALE	unknow	R0	G2	M0	NX	T2	Stage II	unknow
TCGA-DD-A3A0	Tumor	LIHC	785	70	Dead	MALE	WHITE	R0	G2	M0	NX	T1	Stage I	1
TCGA-DD-A3A1	Tumor	LIHC	233	65	Dead	MALE	unknow	R0	G2	M0	N0	T3b	Stage IIIA	6
TCGA-DD-A3A2	Tumor	LIHC	2131	76	Dead	FEMALE	WHITE	R0	G1	M0	N0	T1	Stage I	unknow
TCGA-DD-A3A3	Tumor	LIHC	535	45	Dead	MALE	ASIAN	R0	G2	M0	N0	T1	Stage I	6
TCGA-DD-A3A4	Tumor	LIHC	612	37	Dead	MALE	WHITE	R0	G3	M0	N0	T3	Stage IIIA	unknow
TCGA-DD-A3A5	Tumor	LIHC	3125	66	Dead	FEMALE	WHITE	R0	G2	M0	N0	T3	Stage III	55
TCGA-DD-A3A6	Tumor	LIHC	3258	72	Dead	FEMALE	WHITE	R0	G2	M0	N0	T2	Stage II	unknow
TCGA-DD-A3A7	Tumor	LIHC	419	67	Dead	MALE	unknow	R0	G3	M0	N0	T3b	Stage IIIB	120
TCGA-DD-A3A8	Tumor	LIHC	11	75	Dead	MALE	WHITE	R0	G2	M0	N0	T2	Stage II	4
TCGA-DD-A3A9	Tumor	LIHC	931	64	Dead	FEMALE	WHITE	R0	G2	M1	N0	T4	Stage IVB	19930
TCGA-DD-A4NA	Tumor	LIHC	1008	67	Alive	FEMALE	WHITE	R0	G3	M0	N1	T2	Stage IIIC	3
TCGA-DD-A4NB	Tumor	LIHC	391	25	Alive	MALE	WHITE	R0	G2	M0	N0	T1	Stage I	3
TCGA-DD-A4ND	Tumor	LIHC	2232	56	Alive	FEMALE	WHITE	R0	G3	M0	N0	T1	Stage I	unknow

TCGA-DD-A4NE	Tumor	LIHC	660	75	Dead	FEMALE	WHITE	R0	G3	M0	N0	T3a	Stage IIIA	28
TCGA-DD-A4NF	Tumor	LIHC	428	72	Alive	MALE	WHITE	R0	G2	M0	N0	T1	Stage I	6
TCGA-DD-A4NG	Tumor	LIHC	802	77	Alive	MALE	WHITE	R0	G2	M0	NX	T3a	Stage IIIA	3
TCGA-DD-A4NH	Tumor	LIHC	690	65	Alive	FEMALE	WHITE	R0	G3	M0	N0	T3b	Stage IIIB	92889
TCGA-DD-A4NI	Tumor	LIHC	561	67	Alive	MALE	WHITE	R0	G2	M0	NX	T2	Stage II	6
TCGA-DD-A4NJ	Tumor	LIHC	760	54	Alive	FEMALE	WHITE	R0	G2	M0	N0	T2	Stage II	5
TCGA-DD-A4NK	Tumor	LIHC	1210	80	Dead	FEMALE	WHITE	R0	G2	M0	N0	T3	Stage IIIA	3
TCGA-DD-A4NL	Tumor	LIHC	1711	46	Alive	MALE	WHITE	R0	G1	M0	N0	T1	Stage I	2
TCGA-DD-A4NN	Tumor	LIHC	899	56	Dead	FEMALE	WHITE	R0	G3	M0	N0	T1	Stage I	4223
TCGA-DD-A4NO	Tumor	LIHC	2245	65	Alive	MALE	WHITE	R0	G1	M0	N0	T1	Stage I	3
TCGA-DD-A4NP	Tumor	LIHC	3104	32	Alive	MALE	WHITE	R0	G3	M0	N0	T1	Stage I	2
TCGA-DD-A4NQ	Tumor	LIHC	373	60	Dead	MALE	WHITE	R0	G3	M0	N0	T2	Stage II	141
TCGA-DD-A4NR	Tumor	LIHC	9	85	Dead	FEMALE	WHITE	R0	G3	M0	N0	T1	Stage I	40250
TCGA-DD-A4NS	Tumor	LIHC	2456	61	Dead	FEMALE	WHITE	R0	G2	M0	N0	T1	Stage I	2
TCGA-	Tumor	LIHC	2018	61	Alive	MALE	WHITE	R0	G1	M0	N0	T3	Stage IIIA	3

DD-A4NV														
TCGA-DD-A73A	Tumor	LIHC	728	71	Alive	MALE	WHITE	R0	G2	M0	N0	T1	Stage I	4
TCGA-DD-A73B	Tumor	LIHC	283	72	Dead	FEMALE	WHITE	R0	G2	M0	N0	T1	Stage I	30
TCGA-DD-A73C	Tumor	LIHC	701	65	Alive	FEMALE	WHITE	R0	G1	M0	N0	T3a	Stage IIIA	3
TCGA-DD-A73D	Tumor	LIHC	693	68	Alive	FEMALE	WHITE	R0	G1	MX	NX	T2	Stage II	4
TCGA-DD-A73E	Tumor	LIHC	44	66	Alive	MALE	WHITE	R0	G1	M0	N0	T1	Stage I	1
TCGA-DD-A73F	Tumor	LIHC	1085	77	Alive	FEMALE	WHITE	R0	G1	M0	N0	T1	Stage I	283
TCGA-DD-A73G	Tumor	LIHC	3478	73	Alive	FEMALE	WHITE	R0	G3	M0	N0	T1	Stage I	2035400
TCGA-DD-AA3A	Tumor	LIHC	410	81	Dead	FEMALE	WHITE	R0	G4	MX	N0	T1	Stage I	1
TCGA-DD-AAC8	Tumor	LIHC	16	72	Dead	MALE	ASIAN	R0	G3	M0	N0	T1	Stage I	1
TCGA-DD-AAC9	Tumor	LIHC	347	51	Alive	MALE	ASIAN	R0	G2	M0	N0	T1	Stage I	5
TCGA-DD-AACA	Tumor	LIHC	2301	65	Alive	MALE	ASIAN	R0	G3	M0	N0	T1	Stage I	7
TCGA-DD-AACB	Tumor	LIHC	2324	74	Alive	FEMALE	ASIAN	R0	G3	M0	N0	T1	Stage I	1902
TCGA-DD-AACC	Tumor	LIHC	1685	61	Dead	MALE	ASIAN	R0	G2	M0	N0	T1	Stage I	24
TCGA-DD-AACD	Tumor	LIHC	381	48	Dead	MALE	ASIAN	R0	G4	M0	N0	T1	Stage I	2

TCGA-DD-AACE	Tumor	LIHC	2184	62	Alive	MALE	ASIAN	R0	G3	M0	N0	T1	Stage I	2
TCGA-DD-AACF	Tumor	LIHC	365	68	Dead	MALE	ASIAN	R0	G3	M0	N0	T1	Stage I	16
TCGA-DD-AACG	Tumor	LIHC	469	52	Dead	MALE	ASIAN	R0	G4	M0	N0	T2	Stage II	11718
TCGA-DD-AACH	Tumor	LIHC	195	69	Dead	MALE	ASIAN	R0	G3	M0	N0	T2	Stage II	7
TCGA-DD-AACI	Tumor	LIHC	1618	69	Alive	MALE	ASIAN	R0	G3	M0	N0	T2	Stage II	1
TCGA-DD-AACJ	Tumor	LIHC	2102	75	Alive	MALE	ASIAN	R0	G2	M0	N0	T2	Stage II	25
TCGA-DD-AACK	Tumor	LIHC	9	70	Alive	MALE	ASIAN	R0	G2	M0	N0	T1	Stage I	5
TCGA-DD-AACL	Tumor	LIHC	107	66	Alive	FEMALE	ASIAN	R0	G3	M0	N0	T1	Stage I	1368
TCGA-DD-AACM	Tumor	LIHC	1769	48	Alive	MALE	ASIAN	R0	G3	M0	N0	T2	Stage II	2
TCGA-DD-AACN	Tumor	LIHC	1302	32	Alive	MALE	ASIAN	R0	G3	M0	N0	T1	Stage I	699
TCGA-DD-AACO	Tumor	LIHC	1876	40	Alive	MALE	ASIAN	R0	G3	M0	N0	T1	Stage I	5
TCGA-DD-AACP	Tumor	LIHC	415	64	Alive	MALE	ASIAN	R0	G3	M0	N0	T1	Stage I	227
TCGA-DD-AACQ	Tumor	LIHC	432	50	Alive	MALE	ASIAN	R0	G3	M0	N0	T2	Stage II	7
TCGA-DD-AACS	Tumor	LIHC	1804	39	Alive	MALE	ASIAN	R0	G3	M0	N0	T1	Stage I	1
TCGA-	Tumor	LIHC	1562	69	Alive	FEMALE	ASIAN	R0	G2	M0	N0	T1	Stage I	4

DD-AACT														
TCGA-DD-AACU	Tumor	LIHC	1567	59	Alive	MALE	ASIAN	R0	G3	M0	N0	T1	Stage I	22
TCGA-DD-AACV	Tumor	LIHC	1531	53	Alive	MALE	ASIAN	R0	G3	M0	N0	T1	Stage I	9
TCGA-DD-AACW	Tumor	LIHC	1424	43	Alive	MALE	ASIAN	R0	G3	M0	N0	T1	Stage I	1726
TCGA-DD-AACX	Tumor	LIHC	170	66	Alive	MALE	ASIAN	R0	G3	M0	N0	T2	Stage II	3
TCGA-DD-AACY	Tumor	LIHC	1450	61	Alive	MALE	ASIAN	R0	G3	M0	N0	T1	Stage I	290
TCGA-DD-AACZ	Tumor	LIHC	171	63	Dead	FEMALE	ASIAN	R0	G4	M0	N0	T1	Stage I	25
TCGA-DD-AAD0	Tumor	LIHC	137	73	Alive	FEMALE	ASIAN	R0	G2	M0	N0	T1	Stage I	4
TCGA-DD-AAD1	Tumor	LIHC	564	51	Alive	FEMALE	ASIAN	R1	G4	M0	N0	T1	Stage I	451
TCGA-DD-AAD2	Tumor	LIHC	658	66	Alive	MALE	ASIAN	R0	G2	M0	N0	T1	Stage I	25
TCGA-DD-AAD3	Tumor	LIHC	1295	43	Alive	MALE	ASIAN	R0	G2	M0	N0	T1	Stage I	unknow
TCGA-DD-AAD5	Tumor	LIHC	1345	54	Alive	MALE	ASIAN	R0	G3	M0	N0	T1	Stage I	73
TCGA-DD-AAD6	Tumor	LIHC	672	66	Alive	MALE	ASIAN	R0	G3	M0	N0	T3a	Stage IIIA	unknow
TCGA-DD-AAD8	Tumor	LIHC	1219	73	Alive	FEMALE	ASIAN	R0	G2	M0	N0	T1	Stage I	4
TCGA-DD-AADA	Tumor	LIHC	1233	66	Alive	FEMALE	ASIAN	R0	G3	M0	N0	T1	Stage I	8698

TCGA-DD-AADB	Tumor	LIHC	1242	51	Alive	MALE	ASIAN	R0	G4	M0	N0	T1	Stage I	419
TCGA-DD-AADC	Tumor	LIHC	425	53	Dead	MALE	ASIAN	R0	G3	M0	N0	T1	Stage I	36
TCGA-DD-AADD	Tumor	LIHC	1231	51	Alive	MALE	ASIAN	R0	G4	M0	N0	T1	Stage I	9804
TCGA-DD-AADE	Tumor	LIHC	1202	50	Alive	MALE	ASIAN	R0	G4	M0	N0	T1	Stage I	7
TCGA-DD-AADF	Tumor	LIHC	115	64	Dead	FEMALE	ASIAN	R0	G4	M0	N0	T1	Stage I	725
TCGA-DD-AADG	Tumor	LIHC	1145	70	Alive	MALE	ASIAN	R0	G3	M0	N0	T3a	Stage IIIA	2256
TCGA-DD-AADI	Tumor	LIHC	1085	43	Alive	FEMALE	ASIAN	R0	G3	M0	N0	T1	Stage I	27
TCGA-DD-AADJ	Tumor	LIHC	1066	70	Alive	FEMALE	ASIAN	R0	G3	M0	N0	T1	Stage I	708
TCGA-DD-AADK	Tumor	LIHC	1049	68	Alive	FEMALE	ASIAN	R0	G3	M0	N0	T2	Stage II	236
TCGA-DD-AADL	Tumor	LIHC	636	58	Alive	MALE	ASIAN	R0	G4	M0	N0	T1	Stage I	14
TCGA-DD-AADM	Tumor	LIHC	12	58	Dead	MALE	ASIAN	R0	G3	M0	N0	T2	Stage II	112
TCGA-DD-AADN	Tumor	LIHC	898	59	Alive	MALE	ASIAN	R0	G4	MX	NX	T1	Stage I	151367
TCGA-DD-AADO	Tumor	LIHC	453	55	Alive	MALE	ASIAN	R0	G3	M0	N0	T1	Stage I	176037
TCGA-DD-AADP	Tumor	LIHC	458	45	Alive	MALE	ASIAN	R0	G3	M0	N0	T1	Stage I	9
TCGA-	Tumor	LIHC	436	59	Alive	MALE	ASIAN	R0	G3	M0	N0	T2	Stage II	7

DD-AADQ														
TCGA-DD-AADR	Tumor	LIHC	2028	58	Alive	MALE	ASIAN	R0	G3	M0	N0	T1	Stage I	3
TCGA-DD-AADS	Tumor	LIHC	474	63	Alive	MALE	ASIAN	R0	G2	M0	N0	T1	Stage I	11
TCGA-DD-AADU	Tumor	LIHC	554	60	Alive	MALE	ASIAN	R0	G3	M0	N0	T2	Stage II	52
TCGA-DD-AADV	Tumor	LIHC	574	50	Alive	MALE	ASIAN	R0	G3	M0	N0	T1	Stage I	11
TCGA-DD-AADW	Tumor	LIHC	587	48	Alive	MALE	ASIAN	R0	G3	M0	N0	T1	Stage I	250
TCGA-DD-AADY	Tumor	LIHC	555	55	Alive	FEMALE	ASIAN	R0	G2	M0	N0	T1	Stage I	9
TCGA-DD-AAE0	Tumor	LIHC	555	45	Alive	FEMALE	ASIAN	R0	G4	M0	N0	T3a	Stage IIIA	8751
TCGA-DD-AAE1	Tumor	LIHC	552	52	Alive	MALE	ASIAN	R0	G3	M0	N0	T1	Stage I	2693
TCGA-DD-AAE2	Tumor	LIHC	638	51	Alive	MALE	ASIAN	R0	G3	M0	N0	T1	Stage I	41
TCGA-DD-AAE3	Tumor	LIHC	566	50	Alive	MALE	ASIAN	R0	G2	M0	N0	T1	Stage I	2
TCGA-DD-AAE4	Tumor	LIHC	608	49	Alive	FEMALE	ASIAN	R0	G1	M0	N0	T1	Stage I	7
TCGA-DD-AAE6	Tumor	LIHC	141	59	Alive	FEMALE	ASIAN	R0	G2	M0	N0	T1	Stage I	113
TCGA-DD-AAE7	Tumor	LIHC	644	72	Alive	MALE	ASIAN	R0	G2	M0	N0	T1	Stage I	1
TCGA-DD-AAE8	Tumor	LIHC	664	45	Alive	MALE	ASIAN	R0	G3	M0	N0	T1	Stage I	3

TCGA-DD-AAE9	Tumor	LIHC	722	69	Alive	MALE	ASIAN	R0	G3	M0	N0	T1	Stage I	3
TCGA-DD-AAEA	Tumor	LIHC	575	65	Alive	MALE	ASIAN	R0	G3	M0	N0	T1	Stage I	2
TCGA-DD-AAEB	Tumor	LIHC	478	60	Alive	MALE	ASIAN	R0	G2	M0	N0	T1	Stage I	1
TCGA-DD-AAED	Tumor	LIHC	763	51	Alive	MALE	ASIAN	R0	G3	M0	N0	T1	Stage I	2405
TCGA-DD-AAEE	Tumor	LIHC	810	55	Alive	MALE	ASIAN	R0	G4	M0	N0	T1	Stage I	3
TCGA-DD-AAEG	Tumor	LIHC	719	59	Alive	FEMALE	ASIAN	R0	G3	M0	N0	T1	Stage I	1
TCGA-DD-AAEH	Tumor	LIHC	784	73	Alive	MALE	ASIAN	R0	G2	M0	N0	T1	Stage I	unknow
TCGA-DD-AAEI	Tumor	LIHC	1531	72	Alive	MALE	ASIAN	R0	G2	M0	N0	T1	Stage I	24
TCGA-DD-AAEK	Tumor	LIHC	1067	51	Alive	MALE	ASIAN	R0	G3	M0	N0	T2	Stage II	5166
TCGA-DD-AAVP	Tumor	LIHC	2752	48	Alive	MALE	ASIAN	R0	G1	M0	N0	T1	Stage I	2
TCGA-DD-AAVQ	Tumor	LIHC	2728	38	Alive	MALE	ASIAN	R0	G2	M0	N0	T1	Stage I	1456
TCGA-DD-AAVR	Tumor	LIHC	2513	44	Alive	MALE	ASIAN	R0	G2	M0	N0	T1	Stage I	36
TCGA-DD-AAVS	Tumor	LIHC	1823	56	Alive	MALE	ASIAN	R0	G2	M0	N0	T1	Stage I	50149
TCGA-DD-AAVU	Tumor	LIHC	2202	46	Alive	MALE	ASIAN	R0	G2	M0	N0	T2	Stage II	2
TCGA-	Tumor	LIHC	2455	56	Alive	MALE	ASIAN	R0	G3	M0	N0	T2	Stage II	7

DD-AAVV														
TCGA-DD-AAVW	Tumor	LIHC	2317	35	Alive	MALE	ASIAN	R0	G2	M0	N0	T1	Stage I	927
TCGA-DD-AAVX	Tumor	LIHC	1718	38	Alive	MALE	ASIAN	R0	G2	M0	N0	T2	Stage II	1
TCGA-DD-AAVY	Tumor	LIHC	1970	56	Alive	MALE	ASIAN	R0	G2	M0	N0	T3	Stage IIIA	11
TCGA-DD-AAVZ	Tumor	LIHC	1900	38	Alive	MALE	ASIAN	R0	G2	M0	N0	T1	Stage I	945
TCGA-DD-AAW0	Tumor	LIHC	2015	54	Alive	MALE	ASIAN	R0	G2	M0	N0	T1	Stage I	3
TCGA-DD-AAW1	Tumor	LIHC	1989	55	Alive	MALE	ASIAN	R0	G2	M0	N0	T3	Stage IIIA	4
TCGA-DD-AAW2	Tumor	LIHC	1855	69	Alive	MALE	ASIAN	R0	G2	M0	N0	T1	Stage I	3
TCGA-DD-AAW3	Tumor	LIHC	1633	69	Alive	MALE	ASIAN	R0	G2	M0	N0	T1	Stage I	3
TCGA-ED-A459	Tumor	LIHC	408	47	Alive	MALE	ASIAN	R0	G2	M0	N0	T2	Stage II	267
TCGA-ED-A4XI	Tumor	LIHC	386	58	Alive	MALE	ASIAN	R0	G3	M0	N0	T2	Stage II	573
TCGA-ED-A5KG	Tumor	LIHC	482	60	Alive	FEMALE	ASIAN	R0	G2	M0	N0	T2	Stage II	3177
TCGA-ED-A627	Tumor	LIHC	423	74	Alive	MALE	WHITE	R0	G2	M0	NX	T1	Stage I	unknow
TCGA-ED-A66X	Tumor	LIHC	406	35	Alive	MALE	ASIAN	R0	G3	M0	N0	T3a	Stage IIIA	5840
TCGA-ED-A66Y	Tumor	LIHC	296	51	Alive	FEMALE	ASIAN	R0	G3	M0	N0	T3a	Stage IIIA	12100

TCGA-ED-A7PX	Tumor	LIHC	6	48	Alive	FEMALE	ASIAN	R0	G3	M0	NX	T2	Stage II	24
TCGA-ED-A7PY	Tumor	LIHC	390	20	Alive	FEMALE	ASIAN	R0	G3	M0	NX	T2	Stage II	2
TCGA-ED-A7PZ	Tumor	LIHC	6	61	Alive	MALE	ASIAN	R0	G2	M0	NX	T2	Stage II	2
TCGA-ED-A7XO	Tumor	LIHC	427	29	Alive	MALE	ASIAN	R0	G2	M0	N0	T3a	Stage IIIA	4
TCGA-ED-A7XP	Tumor	LIHC	400	53	Alive	FEMALE	ASIAN	R0	G3	M0	N0	T2	Stage II	18
TCGA-ED-A82E	Tumor	LIHC	408	60	Alive	FEMALE	ASIAN	R0	G2	M0	N0	T3a	Stage IIIA	4
TCGA-ED-A8O5	Tumor	LIHC	406	59	Alive	FEMALE	ASIAN	R0	G3	M0	N0	T3a	Stage IIIA	498
TCGA-ED-A8O6	Tumor	LIHC	56	50	Alive	FEMALE	ASIAN	R0	G3	M0	N0	T3a	Stage IIIA	3000
TCGA-ED-A97K	Tumor	LIHC	6	54	Alive	MALE	ASIAN	R0	G2	M0	N0	T3a	Stage IIIA	3
TCGA-EP-A12J	Tumor	LIHC	330	62	Alive	MALE	BLACK OR AFRICAN AMERICAN	R0	G1	MX	NX	T1	Stage I	unknow
TCGA-EP-A26S	Tumor	LIHC	237	70	Alive	MALE	WHITE	unknow	G2	MX	N0	T1	Stage I	unknow
TCGA-EP-A2KA	Tumor	LIHC	357	52	Alive	FEMALE	WHITE	R0	G3	MX	NX	T3a	Stage IIIA	13
TCGA-EP-A2KB	Tumor	LIHC	334	46	Alive	FEMALE	WHITE	R0	G2	MX	NX	T1	Stage I	2128
TCGA-EP-A2KC	Tumor	LIHC	19	62	Dead	MALE	BLACK OR AFRICAN AMERICAN	R0	G3	MX	NX	T1	Stage I	43
TCGA-	Tumor	LIHC	303	76	Alive	MALE	WHITE	unknow	G2	MX	NX	T1	Stage I	13

EP-A3JL														
TCGA-EP-A3RK	Tumor	LIHC	363	73	Alive	MALE	WHITE	unknow	G2	MX	NX	T3a	Stage IIIA	7
TCGA-ES-A2HS	Tumor	LIHC	688	80	Dead	MALE	WHITE	R0	G2	MX	NX	T1	Stage I	1
TCGA-ES-A2HT	Tumor	LIHC	438	54	Dead	MALE	BLACK OR AFRICAN AMERICAN	R0	G2	MX	NX	T1	Stage I	4
TCGA-FV-A23B	Tumor	LIHC	1852	70	Alive	FEMALE	WHITE	R0	unknow	M0	N0	T2	Stage II	unknow
TCGA-FV-A2QQ	Tumor	LIHC	729	80	Alive	MALE	WHITE	R0	G2	MX	N0	T1	Stage I	unknow
TCGA-FV-A2QR	Tumor	LIHC	581	75	Dead	MALE	WHITE	RX	G1	M0	N0	T1	Stage I	unknow
TCGA-FV-A3I0	Tumor	LIHC	848	76	Alive	FEMALE	WHITE	R0	G2	M0	NX	T2	Stage II	unknow
TCGA-FV-A3I1	Tumor	LIHC	247	81	Alive	FEMALE	WHITE	R0	G2	MX	N0	T2	Stage II	unknow
TCGA-FV-A3R2	Tumor	LIHC	194	75	Dead	MALE	WHITE	R0	unknow	MX	NX	T1	Stage I	unknow
TCGA-FV-A3R3	Tumor	LIHC	366	38	Alive	FEMALE	WHITE	R0	G2	MX	NX	T1	Stage I	unknow
TCGA-FV-A495	Tumor	LIHC	1	51	Alive	FEMALE	WHITE	R0	G2	M0	NX	T2	Stage II	39
TCGA-FV-A496	Tumor	LIHC	10	84	Alive	FEMALE	WHITE	R0	G2	M0	NX	T1	Stage I	67
TCGA-FV-A4ZP	Tumor	LIHC	2486	78	Alive	MALE	WHITE	R1	G2	M0	NX	T3	Stage IIIA	20326
TCGA-FV-A4ZQ	Tumor	LIHC	12	52	Alive	MALE	WHITE	R0	G2	M0	NX	T1	Stage I	unknow

TCGA-G3-A25S	Tumor	LIHC	416	64	Alive	MALE	WHITE	R1	G2	M0	N0	T1	Stage I	281
TCGA-G3-A25T	Tumor	LIHC	1553	45	Alive	FEMALE	WHITE	R0	G2	M0	N0	T3	Stage IIIA	7
TCGA-G3-A25U	Tumor	LIHC	1636	63	Alive	FEMALE	ASIAN	R0	G3	M0	N0	T1	Stage I	10
TCGA-G3-A25V	Tumor	LIHC	860	68	Alive	MALE	WHITE	R0	G2	M0	N0	T1	Stage I	15
TCGA-G3-A25W	Tumor	LIHC	935	79	Alive	FEMALE	WHITE	R0	G2	M0	N0	T3b	Stage IIIB	6
TCGA-G3-A25X	Tumor	LIHC	1779	73	Alive	MALE	ASIAN	R0	G3	M0	N0	T2	Stage II	11
TCGA-G3-A25Y	Tumor	LIHC	452	52	Dead	FEMALE	ASIAN	R0	G3	M0	N0	T1	Stage I	35
TCGA-G3-A25Z	Tumor	LIHC	655	58	Alive	MALE	ASIAN	R0	G2	M0	N0	T1	Stage I	44
TCGA-G3-A3CG	Tumor	LIHC	673	80	Alive	MALE	WHITE	R1	G2	M0	N0	T1	Stage I	67
TCGA-G3-A3CH	Tumor	LIHC	780	53	Alive	MALE	ASIAN	R0	G2	M0	N0	T3a	Stage IIIA	41
TCGA-G3-A3CI	Tumor	LIHC	180	71	Alive	MALE	WHITE	R0	G2	M0	N0	T1	Stage I	2
TCGA-G3-A3CJ	Tumor	LIHC	594	52	Alive	MALE	AMERICAN INDIAN OR ALASKA NATIVE	R0	G2	M0	N0	T2	Stage II	3
TCGA-G3-A3CK	Tumor	LIHC	585	61	Alive	MALE	ASIAN	R0	G2	M0	N0	T1	Stage I	3
TCGA-G3-A5SI	Tumor	LIHC	768	44	Alive	MALE	ASIAN	R0	G2	M0	N0	T2	Stage II	9

TCGA-G3-A5SJ	Tumor	LIHC	698	59	Alive	MALE	WHITE	R0	G2	M0	NX	T1	Stage I	3
TCGA-G3-A5SK	Tumor	LIHC	744	58	Alive	MALE	WHITE	R0	G1	M0	NX	T1	Stage I	unknow
TCGA-G3-A5SL	Tumor	LIHC	621	70	Alive	MALE	WHITE	R0	G2	M0	NX	T2	Stage II	4
TCGA-G3-A5SM	Tumor	LIHC	520	58	Alive	MALE	WHITE	R0	G3	M0	NX	T2	Stage II	12
TCGA-G3-A6UC	Tumor	LIHC	671	65	Alive	MALE	WHITE	R0	G2	M0	N0	T3b	Stage IIIB	4
TCGA-G3-A7M5	Tumor	LIHC	447	76	Alive	MALE	ASIAN	R0	G2	MX	NX	T1	Stage I	6
TCGA-G3-A7M6	Tumor	LIHC	632	60	Alive	FEMALE	WHITE	R0	G3	MX	NX	T1	Stage I	24
TCGA-G3-A7M7	Tumor	LIHC	361	65	Alive	MALE	WHITE	R0	G1	MX	NX	T1	Stage I	4
TCGA-G3-A7M8	Tumor	LIHC	430	31	Alive	MALE	ASIAN	RX	G1	MX	NX	T1	Stage I	5
TCGA-G3-A7M9	Tumor	LIHC	56	70	Dead	MALE	WHITE	R0	G2	MX	NX	T3b	Stage IIIB	22868
TCGA-G3-AAUZ	Tumor	LIHC	480	48	Alive	MALE	unknow	R0	G2	M0	N0	T1	Stage I	3
TCGA-G3-AAV0	Tumor	LIHC	476	58	Alive	MALE	ASIAN	R0	G2	M0	N0	T1	Stage I	4
TCGA-G3-AAV1	Tumor	LIHC	359	51	Dead	MALE	ASIAN	R0	G3	M0	N0	T4	Stage IIIC	8
TCGA-G3-AAV2	Tumor	LIHC	372	50	Alive	MALE	WHITE	R0	G1	M0	N0	T1	Stage I	4
TCGA-	Tumor	LIHC	412	58	Alive	FEMALE	WHITE	R0	G2	M0	N0	T2	Stage II	5

G3-AAV3														
TCGA-G3-AAV4	Tumor	LIHC	27	83	Dead	FEMALE	WHITE	R0	G1	M0	N0	T1	Stage I	4
TCGA-G3-AAV5	Tumor	LIHC	354	67	Alive	MALE	WHITE	R0	G2	M0	N0	T2	Stage II	unknow
TCGA-G3-AAV6	Tumor	LIHC	65	53	Dead	FEMALE	WHITE	R0	G3	M0	N0	T3a	Stage IIIA	57875
TCGA-G3-AAV7	Tumor	LIHC	361	38	Alive	MALE	ASIAN	R0	G2	M0	N0	T2	Stage II	3
TCGA-GJ-A3OU	Tumor	LIHC	879	59	Alive	MALE	WHITE	RX	G2	MX	NX	T1	Stage I	8
TCGA-GJ-A6C0	Tumor	LIHC	31	75	Dead	FEMALE	WHITE	RX	G2	MX	NX	T2	Stage II	unknow
TCGA-GJ-A9DB	Tumor	LIHC	67	68	Dead	MALE	WHITE	R0	G2	MX	N0	T1	Stage I	unknow
TCGA-HP-A5MZ	Tumor	LIHC	91	78	Dead	MALE	unknow	R0	G2	M0	NX	T1	Stage I	unknow
TCGA-HP-A5N0	Tumor	LIHC	752	90	Dead	FEMALE	unknow	R0	unknow	M0	NX	TX	unknow	unknow
TCGA-K7-A5RF	Tumor	LIHC	631	64	Alive	MALE	WHITE	R0	G1	MX	NX	T1	Stage I	5
TCGA-K7-A5RG	Tumor	LIHC	519	66	Alive	MALE	BLACK OR AFRICAN AMERICAN	R0	G1	MX	NX	T1	Stage I	19
TCGA-K7-A6G5	Tumor	LIHC	512	66	Alive	MALE	WHITE	RX	G2	MX	N0	T1	Stage I	unknow
TCGA-K7-AAU7	Tumor	LIHC	359	61	Alive	MALE	WHITE	RX	G2	MX	NX	T2a	Stage II	290
TCGA-KR-A7K0	Tumor	LIHC	65	65	Dead	MALE	WHITE	R0	G1	M0	N0	T1	Stage I	11

TCGA-KR-A7K2	Tumor	LIHC	657	64	Alive	MALE	WHITE	R0	G1	M0	N0	T1	Stage I	14
TCGA-KR-A7K7	Tumor	LIHC	407	61	Alive	FEMALE	WHITE	R0	G1	M0	N0	T2	Stage II	308836
TCGA-KR-A7K8	Tumor	LIHC	906	57	Alive	MALE	unknow	R0	G1	M0	N0	T1	Stage I	154
TCGA-LG-A6GG	Tumor	LIHC	387	79	Alive	FEMALE	WHITE	R0	G2	M0	NX	T2	Stage II	unknow
TCGA-LG-A9QC	Tumor	LIHC	425	48	Alive	MALE	WHITE	R0	G2	M0	NX	T1	Stage I	unknow
TCGA-LG-A9QD	Tumor	LIHC	366	68	Alive	MALE	WHITE	R0	G2	M0	N0	T3a	Stage IIIA	unknow
TCGA-MI-A75C	Tumor	LIHC	291	64	Alive	MALE	WHITE	R0	G3	M0	N0	T1	Stage I	4
TCGA-MI-A75E	Tumor	LIHC	507	61	Alive	MALE	WHITE	R0	G2	M0	N0	T4	Stage IIIC	5
TCGA-MI-A75G	Tumor	LIHC	698	63	Alive	MALE	WHITE	R0	G2	M0	N0	T2	Stage II	6
TCGA-MI-A75H	Tumor	LIHC	747	77	Alive	MALE	WHITE	R0	unknow	MX	NX	unknow	unknow	11
TCGA-MI-A75I	Tumor	LIHC	630	61	Alive	MALE	BLACK OR AFRICAN AMERICAN	R0	G1	MX	NX	T2	unknow	unknow
TCGA-MR-A520	Tumor	LIHC	229	58	Alive	MALE	WHITE	unknow	G1	MX	NX	T1	Stage I	unknow
TCGA-MR-A8JO	Tumor	LIHC	330	34	Alive	MALE	WHITE	RX	G3	MX	N0	T1	Stage I	2
TCGA-NI-A4U2	Tumor	LIHC	1791	71	Dead	MALE	WHITE	R0	G1	MX	NX	T3	Stage IIIA	5
TCGA-	Tumor	LIHC	606	74	Alive	MALE	WHITE	R0	G3	MX	NX	T1	Stage I	unknow

NI-A8LF														
TCGA-O8-A75V	Tumor	LIHC	538	54	Alive	MALE	unknow	R1	G2	MX	NX	T1	Stage I	7
TCGA-PD-A5DF	Tumor	LIHC	639	58	Dead	FEMALE	WHITE	unknow	G2	M0	N0	T4	Stage IIIB	unknow
TCGA-QA-A7B7	Tumor	LIHC	94	48	Alive	MALE	BLACK OR AFRICAN AMERICAN	RX	G2	MX	NX	T2	Stage II	1751
TCGA-RC-A6M3	Tumor	LIHC	unknow	24	Alive	MALE	AMERICAN INDIAN OR ALASKA NATIVE	R0	G3	M0	N0	T2	Stage II	unknow
TCGA-RC-A6M4	Tumor	LIHC	22	74	Alive	FEMALE	WHITE	R1	G2	MX	NX	T3	Stage IIIA	5
TCGA-RC-A6M5	Tumor	LIHC	15	20	Alive	FEMALE	WHITE	R0	G2	M0	N1	T1	Stage IVA	unknow
TCGA-RC-A6M6	Tumor	LIHC	9	75	Alive	MALE	WHITE	R1	G3	M0	NX	T2	Stage II	12986
TCGA-RC-A7S9	Tumor	LIHC	640	47	Alive	FEMALE	ASIAN	R0	G3	M0	N0	T1	Stage I	10
TCGA-RC-A7SB	Tumor	LIHC	588	53	Alive	MALE	ASIAN	R0	G2	M0	N0	T2	Stage II	3
TCGA-RC-A7SF	Tumor	LIHC	579	66	Alive	MALE	ASIAN	R0	G2	M0	N0	T1	Stage I	87
TCGA-RC-A7SH	Tumor	LIHC	468	42	Alive	MALE	ASIAN	R0	G3	M0	N0	T2	Stage II	2466
TCGA-RC-A7SK	Tumor	LIHC	472	59	Alive	MALE	ASIAN	R0	G3	M0	N0	T1	Stage I	25
TCGA-RG-A7D4	Tumor	LIHC	1098	69	Alive	MALE	BLACK OR AFRICAN AMERICAN	R0	G2	M0	N0	T2	Stage II	5650
TCGA-	Tumor	LIHC	23	68	Alive	MALE	WHITE	RX	G2	M0	NX	T1	unknow	unknow

T1-A6J8														
TCGA-UB-A7MA	Tumor	LIHC	535	62	Alive	FEMALE	WHITE	R1	G2	M0	N0	T2b	Stage II	2008
TCGA-UB-A7MB	Tumor	LIHC	601	24	Alive	MALE	WHITE	R0	G3	MX	NX	T2	Stage II	1865
TCGA-UB-A7MC	Tumor	LIHC	500	59	Alive	MALE	WHITE	R0	G3	MX	N0	T3a	Stage IIIA	126
TCGA-UB-A7MD	Tumor	LIHC	52	67	Dead	MALE	BLACK OR AFRICAN AMERICAN	R0	G3	MX	N0	T1	Stage I	23
TCGA-UB-A7ME	Tumor	LIHC	486	51	Alive	MALE	ASIAN	R0	G2	MX	NX	T1	Stage I	1388
TCGA-UB-A7MF	Tumor	LIHC	214	56	Dead	MALE	WHITE	R0	G2	MX	NX	T3a	Stage IIIA	2505
TCGA-UB-AA0U	Tumor	LIHC	327	60	Alive	MALE	WHITE	R1	G2	MX	NX	T2	Stage II	26
TCGA-UB-AA0V	Tumor	LIHC	314	69	Alive	FEMALE	WHITE	R0	G1	MX	NX	unknow	Stage I	3
TCGA-WJ-A86L	Tumor	LIHC	345	68	Alive	FEMALE	WHITE	R0	G2	MX	NX	T1	Stage I	16
TCGA-WQ-A9G7	Tumor	LIHC	30	71	Alive	FEMALE	WHITE	R0	G3	M0	NX	T3a	unknow	unknow
TCGA-WQ-AB4B	Tumor	LIHC	395	62	Alive	MALE	WHITE	R0	G2	M0	NX	T2	Stage II	5
TCGA-WX-AA44	Tumor	LIHC	615	64	Alive	FEMALE	WHITE	RX	G3	MX	NX	T1	Stage I	48
TCGA-WX-AA46	Tumor	LIHC	756	61	Alive	MALE	WHITE	R0	G1	MX	NX	T2	Stage II	1
TCGA-WX-AA47	Tumor	LIHC	556	33	Dead	FEMALE	WHITE	R0	G2	MX	NX	T3a	Stage IIIA	1

TCGA-XR-A8TC	Tumor	LIHC	1339	43	Alive	FEMALE	WHITE	R0	G2	MX	NX	T1	Stage I	47
TCGA-XR-A8TD	Tumor	LIHC	1030	49	Alive	FEMALE	WHITE	R0	G3	M0	N0	T3	Stage IIIB	188
TCGA-XR-A8TE	Tumor	LIHC	925	16	Alive	MALE	WHITE	R0	G1	MX	N0	T3	Stage IIIA	809
TCGA-XR-A8TF	Tumor	LIHC	693	74	Dead	MALE	WHITE	R0	G1	MX	NX	T1	Stage I	39
TCGA-XR-A8TG	Tumor	LIHC	898	58	Alive	MALE	WHITE	R0	G2	M0	NX	T1	Stage I	1
TCGA-YA-A8S7	Tumor	LIHC	412	68	Dead	MALE	WHITE	R0	G3	MX	N0	T3a	Stage IIIA	56
TCGA-ZP-A9CV	Tumor	LIHC	1088	59	Alive	MALE	WHITE	R0	G1	MX	NX	T1	unknow	48
TCGA-ZP-A9CY	Tumor	LIHC	782	66	Alive	FEMALE	WHITE	R0	G1	MX	NX	T1	unknow	unknow
TCGA-ZP-A9CZ	Tumor	LIHC	706	72	Alive	MALE	ASIAN	R0	G1	MX	NX	T1	unknow	8
TCGA-ZP-A9D0	Tumor	LIHC	717	67	Alive	FEMALE	BLACK OR AFRICAN AMERICAN	R0	G1	MX	NX	T1	unknow	unknow
TCGA-ZP-A9D1	Tumor	LIHC	21	56	Alive	FEMALE	WHITE	R0	G2	MX	NX	T1	unknow	8
TCGA-ZP-A9D2	Tumor	LIHC	743	51	Alive	MALE	WHITE	R0	G2	MX	NX	T2	unknow	unknow
TCGA-ZP-A9D4	Tumor	LIHC	395	64	Alive	FEMALE	WHITE	R0	G1	MX	NX	T1	unknow	2
TCGA-ZS-A9CD	Tumor	LIHC	1386	73	Dead	MALE	WHITE	R0	G2	MX	NX	T2	Stage II	unknow
TCGA-	Tumor	LIHC	1241	79	Alive	FEMALE	WHITE	R0	G1	MX	NX	T2	Stage II	unknow

ZS-<br>A9CE														
TCGA-<br>ZS-<br>A9CF	Tumor	LIHC	2412	64	Alive	MALE	WHITE	R0	G2	MX	NX	T2	Stage II	unknow
TCGA-<br>ZS-<br>A9CG	Tumor	LIHC	341	55	Alive	MALE	WHITE	R0	G2	MX	NX	T2	Stage II	unknow

Table S2. TCGA liver cancer patient characteristics			
**Characteristic**	**Low expression of AL355574.1**	**High expression of AL355574.1**	**P**
n	187	187	
**T-stage, n (%)**			**0.014**
T1	105(28.5%)	76(20.7%)	
T2	38(10.3%)	56(15.2%)	
T3	35(9.5%)	45(12.2%)	
T4	7(1.9%)	6(1.6%)	
**N-stage, n (%)**			0.95
N0	122(47.7%)	130(50.8%)	
N1	2(0.8%)	2(0.8%)	
**M-stage, n (%)**			0.266
M0	125(46.3%)	141(52.5%)	
M1	3(1.1%)	1(0.4%)	
**Pathologic Stage, n (%)**			**0.024**
Stage I	99(28.5%)	72(20.7%)	
Stage II	35(10.1%)	51(14.7%)	
Stage III	37(10.7%)	48(13.8%)	
Stage IV	4(1.2%)	1(0.3%)	
**Histological Grade, n (%)**			**0**
G1	38(10.4%)	17(4.6%)	
G2	94(25.7%)	83(22.7%)	
G3	46(12.6%)	76(20.8%)	
G4	6(1.6%)	6(1.6%)	
**Gender, n (%)**			0.27
Female	56(15.1%)	65(17.5%)	
Male	131(35.3%)	119(32.1%)	
**Age, n(%)**			0.708
<=65	119(32.2%)	113(30.5%)	
>65	68(18.4%)	70(18.9%)	
**Cancer status, n (%)**			0.223
tumor free	125(36.3%)	109(31.7%)	
with tumor	51(14.8%)	59(17.2%)	
**Residual tumor, n (%)**			0.449
R0	164(48.1%)	160(46.9%)	
R1	7(2.1%)	10(2.9%)	
**History hepato carcinoma risk factors, n (%)**			**0.042**
Alcohol consumption	27(16.3%)	38(22.9%)	
Hepatitis B	38(22.9%)	35(21.1%)	
Hepatitis C	18(10.8%)	10(6.0%)	

Table S3. AL355574.1 expression correlated with clinical pathological characteristics (logistic regression)

Characteristics	Total(N)	Odds Ratio (OR)	p value
T stage (T4 vs. T1)	355	1.912(1.256-2.922)	**0.00259**
N stage (N1 vs. N0)	256	1 (0.118–8.440)	1
M stage (M1 vs. M0)	270	0.328(0.016–2.601)	0.338
Pathological stage<br>(Stage III & Stage II vs. Stage I)	342	1.891 (1.233–2.913)	**0.0036**
Histological grade (G3 vs. G1)	177	3.396(1.756-6.764)	**0.00036**
Age (>65 vs <=65)	370	1.047(0.687-1.597)	8.30E-01
Gender (Female vs Male)	371	0.795(0.514-1.228)	0.302
Residual tumour (R1 vs. R0)	341	1.464(0.549-4.121)	4.50E-01
Tumor status<br>(with tumour vs. tumour-free)	344	1.239(0.787–1.955)	0.355
History hepato carcinoma risk factors<br>(Alcohol consumption.<br>vs Hepatitis C)	91	0.395(0.153-0.972)	**0.046**

Table S4. Univariate analysis and multivariate analyses of liver cancer patient overall survival

Characterical	Univariate analysis		Multivariate analysis	
	**Hazard ratio(95%Cl)**	**p value**	**Hazard ratio(95%Cl)**	**p value**
T stage (T2&T3&T4 vs. T1)	1.804(1.434-2.270)	**4.73E-07**	1.592(0.678-3.738)	0.285524
N stage (N1 vs. N0)	2.021(0.494-8.276)	0.327563	2.753(0.454-16.703)	0.270837
Pathological stage(Stage Ⅱ &stageⅢ &Stage Ⅳ vs. stage Ⅰ)	1.865(1.456-2.388)	**8.07E-07**	1.090(0.423-2.809)	0.858182
Age (>65 vs.≤ 65 years)	1.005(0.987-1.023)	0.591	1.009(0.989-1.028)	0.387763
Gender (male vs. female)	0.780(0.487-1.249)	0.301	1.075(0.642-1.800)	0.78289
Histological grade （G2&G3&G4 vs.G1）	1.017(0.746-1.387)	0.914	1.063(0.757-1.494)	0.723705
M stage (M1 vs. N0)	3.850(1.207-12.281)	**0.02275**	2.489(0.625-9.903)	0.195672
AL355574.1 (high vs. low)	2.009(1.415-2.850)	**9.41E-05**	1.990(1.384-2.860)	**0.0002**
